# Interconnected roles of cellular senescence and the immune microenvironment in tumor progression: from pan-cancer mechanisms to glioblastoma implications

**DOI:** 10.1038/s41420-026-03284-8

**Published:** 2026-08-12

**Authors:** Wenwen Zhao, Pengfei Zhang, Lixin Li, Jingran Yang, Jia Cheng, Wenjing Zhou, Yongchao Zhang, Dong Lin, Feng Ding, Jianxiong Ji, Jianyang Du

**Affiliations:** 1 Shandong Provincial Hospital Affiliated to Shandong First Medical University, Jinan, China; 2 Tumor Research and Therapy Center, Shandong Provincial Hospital, Cheeloo College of Medicine, Shandong University, Jinan, China; 3 Jinan Maternity and Child Care Hospital Affiliated to Shandong First Medical University, Jinan, 250021 China; 4 East China Normal University, Shanghai, China; 5 Zhongshan Hospital of Xiamen University, School of Medicine, Xiamen University, Xiamen, 361001 Fujian China; 6 Department of Immunology, School of Clinical and Basic Medical Sciences, Shandong First Medical University, Jinan, China; 7 Department of Neurosurgery, the Second Affiliated Hospital of Zhejiang University School of Medicine, Key Laboratory of Precise Treatment and Clinical Translational Research of Neurological Diseases, Hangzhou, China

**Keywords:** Oncogenesis, Immune cell death

## Abstract

Cellular senescence is an irreversible cell state induced by diverse stressors, characterized by permanent cell cycle arrest coupled with sustained metabolic activity. In the context of tumor evolution, senescence exhibits remarkable complexity and dynamism: it functions not only as an early tumor-suppressive barrier that blocks malignant transformation but also recruits and reprograms immune cells through the senescence-associated secretory phenotype (SASP), thereby constructing a distinctive tumor microenvironment (TME). Concurrently, the status of the immune system, including the efficacy of immunosurveillance and the occurrence of local immune exhaustion, reciprocally determines the ultimate fate of senescent cells and governs the transition between their tumor-suppressive and tumor-promoting effects. This dynamic tripartite regulatory network comprising senescence, immunity, and the tumor constitutes the core framework for understanding tumor heterogeneity and drug resistance mechanisms. Advances in single-cell and spatial multi-omics technologies have provided unprecedented precision for dissecting the spatiotemporal evolution of this network. Current efforts to integrate senescence-targeted interventions, such as combinatorial “induce-modulate-eliminate” strategies, with modern immunotherapy show immense potential, although precisely breaching physical barriers and eradicating highly invasive senescent clones remain formidable challenges. This review systematically examines the molecular mechanisms of cellular senescence, with emphasis on the intrinsic logic governing bidirectional remodeling between senescent cells and the immune microenvironment. Glioblastoma (GBM) serves as the focal disease model throughout, providing in-depth analysis of how these mechanisms manifest within a highly immune-privileged ecosystem and their therapeutic implications. By dissecting the interplay between pan-cancer mechanisms and the GBM-specific microenvironment, this review aims to provide a new theoretical foundation for next-generation precision cancer immunotherapy.

**Figure:**
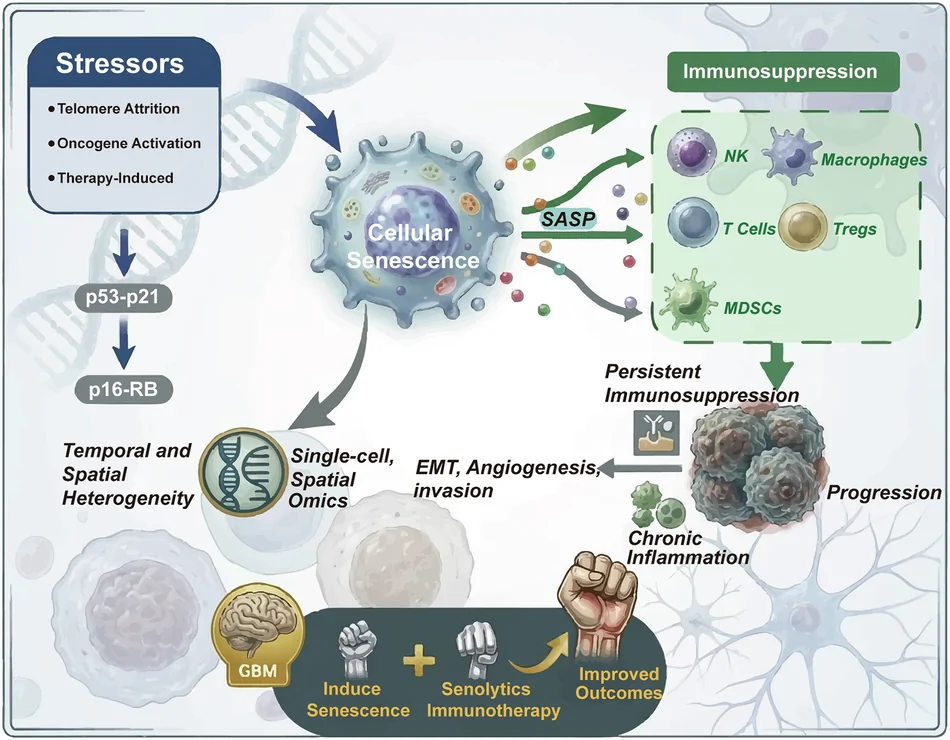

## Facts

- Cellular senescence functions as a double-edged sword in cancer: it suppresses early tumorigenesis through growth arrest but promotes tumor progression, immune evasion, and recurrence through chronic SASP accumulation.
- The senescence–immunity–tumor tripartite network is spatiotemporally dynamic, with acute senescence enhancing immunosurveillance and chronic senescence driving immunosuppression and immune cell exhaustion.
- Therapy-induced senescence (TIS) from standard chemoradiation paradoxically fuels tumor recurrence through senescence escape and stemness reprogramming, particularly in glioblastoma.
- Novel immune checkpoints on senescent cells (B7-H3, VISTA, HLA-E/NKG2A) represent promising targets beyond conventional PD-1/PD-L1 blockade for senescence-rich tumors.
- The “induce–modulate–eliminate” strategy integrating senolytics, senomorphics, and immunotherapy offers a transformative framework for disrupting senescence-driven pro-tumorigenic niches.

## Introduction

Cellular senescence is an irreversible cell state induced by various stressors, including telomere attrition, DNA damage, oxidative stress, and oncogene activation. It was initially characterized as a defense mechanism preventing the proliferation of damaged or potentially malignant cells. However, during tumorigenesis and progression, cellular senescence exhibits remarkable complexity and spatiotemporal dynamism: while it suppresses early tumor formation by halting aberrant cell proliferation, its chronic accumulation within the microenvironment proves profoundly detrimental. If not cleared promptly by the immune system, persistently present senescent cells secrete a repertoire of pro-inflammatory cytokines, growth factors, and matrix-remodeling enzymes, collectively termed the senescence-associated secretory phenotype (SASP). This highly heterogeneous secretory profile profoundly reshapes the tumor microenvironment (TME), thereby promoting tumor progression and inducing deep immune evasion [1, 2].

In recent years, the rapid development of single-cell RNA sequencing (scRNA-seq) and spatial multi-omics has provided unprecedented precision for dissecting the heterogeneity of senescent cells within tissues and tumors. These technologies have revealed that senescence is not merely a static cellular endpoint but rather a highly plastic evolutionary spectrum. Through their SASP factors, specific subpopulations of senescent cells can spatially establish “senescence–immune microdomains,” recruiting and reprogramming immune cells and stromal components, and fundamentally altering the physicochemical properties of the extracellular matrix (ECM). These changes collectively create ecological niches favorable for tumor growth, metastasis, and therapeutic resistance [3–5].

Within these niches, the bidirectional interaction between senescent cells and the immune system constitutes the core force shaping the tumor immune landscape. Under physiological conditions, a healthy immune system, through the coordinated action of natural killer (NK) cells, macrophages, and T cells, efficiently surveys and eliminates senescent cells to maintain tissue homeostasis. However, under prolonged tumor burden, local immunosurveillance is not only attenuated, but senescent cells can also actively reprogram infiltrating immune cells, inducing localized metabolic exhaustion and immunosenescence. This tripartite dynamic regulatory network comprising senescence, immunity, and the tumor is a critical driving force behind persistent inflammation, immunosuppression, and tumor recurrence [6, 7]. Elucidating the interactive logic within this tripartite network is essential for developing strategies that target senescence and enhance cancer immunotherapy.

Although utilizing chemotherapy or radiotherapy to induce tumor cells into senescence, termed therapy-induced senescence (TIS), holds substantial initial therapeutic potential, the persistent survival of residual senescent clones often becomes a covert source of tumor recurrence. Consequently, precision strategies targeting senescence are undergoing a paradigm shift: from mere senescence induction toward a combinatorial approach integrating the modulation of senescent secretions (senomorphics) with the selective elimination of senescent cells (senolytics and novel targeted immunotherapies). This “one-two punch” strategy represents a highly promising frontier for dismantling pro-tumorigenic niches and improving the prognosis of refractory cancers [8].

This review systematically examines the molecular mechanisms of cellular senescence and their complex roles within the TME, with a particular focus on dissecting the deep interactive logic of the senescence–immunity–tumor tripartite network. To integrate pan-cancer mechanisms with specific clinical challenges, glioblastoma (GBM) is adopted as the primary disease model and integrative focus throughout. This approach enables in-depth elucidation of how the senescence–immune network drives cancer progression within the highly immune-privileged central nervous system (CNS) ecosystem, and how multidimensional combinatorial interventions targeting this network may yield new insights for improving patient outcomes. Distinct from existing senescence–immunity reviews that treat senescence as a monolithic state, this review is organized around three integrative axes: (i) it systematically separates tumor cell-intrinsic senescence from senescence of immune and stromal compartments as functionally and therapeutically distinct entities; (ii) it uses spatiotemporal single-cell and spatial multi-omics as the unifying lens that resolves where and when these compartments interact; and (iii) it stress-tests the resulting framework in glioblastoma, the prototypical immune-privileged tumor in which these interactions are most extreme and most therapeutically consequential.

## Mechanisms of cellular senescence and their role in tumorigenesis and cancer progression

### Fundamental mechanisms of cellular senescence

**Fig. 1:**
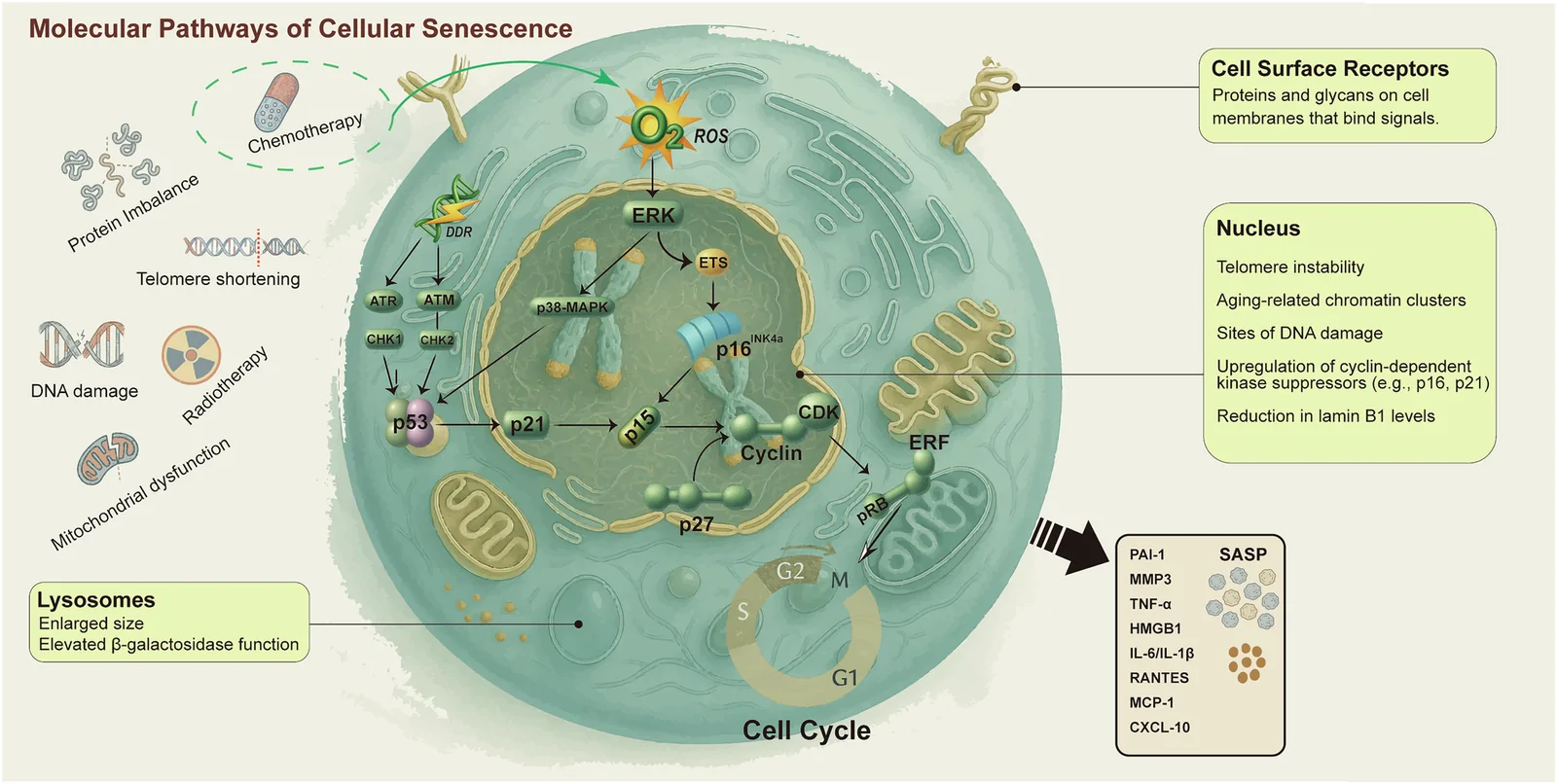
Molecular pathways of cellular senescence. This figure illustrates the key molecular pathways involved in cellular senescence. Various factors, including DNA damage, telomere shortening, mitochondrial dysfunction, and chemotherapy, trigger senescence through different cellular mechanisms. Key players like p53, p21, and p16 regulate cell cycle arrest, while ROS and ERK mediate stress responses. The figure also highlights changes in cellular components such as the nucleus, lysosomes, and cell-surface receptors, contributing to senescence. SASP is also depicted with key molecules such as PAI-1, MMP3, and TNF-α involved in the process.

Cellular senescence is an irreversible cell state triggered by diverse intrinsic and extrinsic stressors, characterized by permanent cell cycle arrest with retained metabolic activity. Key inducers include telomere attrition, DNA damage, reactive oxygen species (ROS) accumulation, proteostasis collapse, mitochondrial dysfunction, and aberrant oncogene activation [9–13] (Fig. 1). These stress signals activate the DNA damage response (DDR), which engages critical cell cycle regulatory networks, particularly the p53–p21 and p16^INK4a^–retinoblastoma protein (RB) pathways, ultimately enforcing G₁/S arrest and loss of proliferative capacity [14]. DDR-mediated telomere erosion and deprotection trigger chromosomal instability and activate ATM/ATR–CHK2/CHK1–p53 signaling cascade, enforcing cell cycle blockade through p21 [14]. Concurrently, p16^INK4a^ inhibits CDK4/6, maintaining RB in a stable hypophosphorylated state to sustain growth arrest [15, 16]. Together, these pathways commit cells to senescence, halting proliferation while preserving cellular viability. Senescent cells also undergo extensive epigenetic remodeling, forming senescence-associated heterochromatic foci (SAHF) that reinforce the silencing of pro-proliferative genes [17].

At the molecular level, the consolidation of this cell state depends not only on cell cycle arrest and epigenetic restructuring but is also established upon profound metabolic reprogramming and homeostatic rebuilding [18]. Mitochondrial damage and sustained overproduction of ROS constitute critical stressors maintaining the senescent state. Meanwhile, persistent mTOR activation suppresses autophagic flux, leading to the intracellular accumulation of damaged proteins and organelles. More importantly, mTOR hyperactivity greatly enhances protein synthesis and secretion, thereby providing abundant material substrates for the subsequent large-scale release of SASP factors. To meet these abnormally increased bioenergetic and biosynthetic demands, the energy metabolism of senescent cells undergoes a marked shift, tilting from glycolysis toward fatty acid oxidation and oxidative phosphorylation [19]. These interwoven metabolic switches and signaling reconfigurations collectively form a complex homeostatic system that firmly locks the senescent phenotype in place.

Within the clinical landscape of tumor biology, senescence is no longer viewed merely as a terminus of cell growth but has emerged as a central point of contention during malignant transformation and therapeutic intervention. Accordingly, oncogene-induced senescence (OIS) and therapy-induced senescence (TIS) have become pivotal areas of precision oncology research. During the early stages of malignant transformation, OIS occurs when oncogenes such as *RAS*, *BRAF*, or *MYC* are aberrantly activated, resulting in permanent cell cycle arrest that prevents malignant conversion [20–22]. OIS described in the HRAS^V12^ model demonstrates that, despite persistent mitogenic signaling, severe replicative stress and DDR activation of the p53–p21 and p16^INK4a^–RB axes lead to irreversible growth arrest and SASP factor secretion [11, 17]. As an early tumor-suppressive mechanism, OIS constrains the expansion of oncogene-bearing cells while enhancing immunosurveillance through pro-inflammatory SASP mediators [23]. In the context of glioma research, OIS holds particular significance. The *BRAF* V600E mutation, frequently observed in low-grade gliomas, often initially provokes a robust OIS response, accounting for the slower progression rate of these tumors. However, when this senescence barrier collapses due to subsequent loss of p53 or *CDKN2A*, tumors rapidly progress toward high-grade glioblastoma [23, 24]. This dynamic equilibrium between senescence induction and escape constitutes a mechanistic foundation for early GBM pathogenesis.

TIS refers to the entry into senescence by multiple cell types within the local tumor environment, including cancer cells, stromal cells, and immune cells, as a response to chemotherapy, radiotherapy, or targeted therapy through intense DNA damage, oxidative stress, or pathway inhibition, rather than through apoptosis alone [25–27]. Chemotherapeutic agents engage the same ATM/ATR–CHK–p53 cascade described above to trigger DNA damage-induced senescence [7]. Temozolomide (TMZ), for instance, induces O⁶-methylguanine lesions in glioma cells, activating ATR–CHK1 signaling and sustained p21 expression, thereby driving prolonged cell cycle arrest and the senescent phenotype [7]. Other agents, including topoisomerase I/II inhibitors, platinum-based compounds, and microtubule inhibitors, similarly induce senescence through DNA damage and mitotic arrest [28–32]. Radiation therapy induces irreparable DNA damage, activating the DDR and senescence [33, 34]. Although localized radiotherapy can spare normal tissues, it may promote the accumulation of senescent cells in adjacent stromal regions, fueling inflammation and immunosuppression. Notably, some senescent cells can escape this arrest, particularly upon loss of p53 or RB, leading to recurrence [35].

While TIS shares common features with OIS, including irreversible arrest, retained metabolism, and SASP secretion, it affects multiple cell types within the TME, including stromal and endothelial cells. Initially, TIS serves as an antitumor mechanism by suppressing proliferation and genomic instability [36, 37], and can enhance therapeutic efficacy, as demonstrated by CDK4/6 inhibitors delaying the progression of dedifferentiated liposarcoma [38]. However, the chronic accumulation of senescent cells and their SASP may paradoxically promote tumor recurrence, inflammation, and tissue dysfunction. In GBM clinical management, this paradox is particularly pronounced. Although the standard Stupp regimen (surgery combined with radiotherapy and TMZ) effectively induces tumor cell senescence, these persistently surviving senescent tumor cells can transition into a “senescent stem cell-like” state and remodel the brain parenchymal microenvironment through SASP [39]. This chronically accumulated senescent state not only provides a nutrient-rich milieu for local tumor infiltration but also induces surrounding astrocytes into a similar senescent state, collectively constructing an immunosuppressive niche supportive of recurrence [40]. Emerging strategies are currently being explored, including the use of senolytics to eliminate TIS cells and senomorphic agents to modulate the deleterious aspects of SASP while preserving therapeutic benefits. Preclinical models indicate that clearing residual senescent tumor cells after chemotherapy can significantly reduce recurrence and metastasis, offering new avenues for improving cancer treatment efficacy [41].

Although senescence has traditionally been considered irreversible, cells can occasionally escape senescence under conditions such as p53 or p16^INK4a^ loss, re-entering the cell cycle and increasing the risk of recurrence [42–45]. This phenomenon of senescence escape enhances chromosomal instability and mutational burden, leading to aggressive tumor relapse [46].

### Hallmarks of senescence and the senescence-associated secretory phenotype

Entry into the senescent state does not represent a simple functional “shutdown” but rather constitutes a profound and complex molecular landscape remodeling. Identifying senescent cells remains a principal challenge in senescence research. Senescence itself is a complex and dynamic process whose hallmark features, such as the DDR, often resemble those of other cellular states, including quiescence, terminal differentiation, dormancy, and drug tolerance [47]. Distinguishing among these states can therefore prove difficult. To date, no universal “gold standard” exists that definitively separates senescent cells from these phenotypically similar states, meaning that identification relies on a panel of nonspecific biomarkers, complicating precise detection [47]. Accurately identifying senescent cells and distinguishing them from other cells exhibiting similar phenotypes has thus become a critical objective for clinical and translational research. Common senescence markers include elevated senescence-associated β-galactosidase (SA-β-gal) activity, increased expression of p16^INK4a^, p21^CIP1^, and p53, SAHF formation, lipofuscin accumulation, increased DNA damage foci, and reduced lamin B1 levels [47, 48]. Recent studies have also explored specific microRNA signatures and satellite DNA expansion as potential markers, although their sensitivity and specificity require further validation. However, these markers exhibit notable dynamism and limitations at the pan-cancer level. For instance, p16^INK4a^ can be compensatorily overexpressed in certain RB-deficient proliferating cancer cells, and SA-β-gal may yield false positives in confluent cell cultures or in macrophages with high autophagic activity. Furthermore, senescence marker emergence is highly time-dependent, and not all senescent cells synchronously express the full panel. In the context of GBM, marker identification presents additional complexity. TMZ commonly induces marked p21 upregulation, yet studies have found that a subset of GBM cells undergoes “p21 leakage,” resulting not in permanent arrest but instead in genomic re-replication and evolution into highly invasive polyploid giant cells [49]. This dynamic fluctuation in marker expression underscores the need to incorporate dynamic indicators such as metabolic reprogramming for more precise delineation of the senescent state within complex tumor microenvironments.

As the core mechanism through which senescent cells exert microenvironmental remodeling, SASP is a hallmark of senescent cells, composed of pro-inflammatory cytokines, growth factors, chemokines, and matrix metalloproteinases (MMPs) [50]. Notably, SASP components are not exclusive to senescent cells; certain non-senescent cells, such as highly activated immune cells or stressed stromal cells, can secrete similar factors under specific microenvironmental pressures, further complicating the precise identification of senescent cells based solely on secretory profiles. SASP plays a highly complex and dynamic role within the tumor ecosystem: on one hand, it can transiently promote immune-mediated clearance of potentially oncogenic cells, but when secreted excessively and persistently, it drives chronic inflammation, induces epithelial-mesenchymal transition (EMT), promotes angiogenesis, and thereby comprehensively accelerates tumor progression and tissue degeneration [3, 4]. Canonical SASP components such as interleukin-6 (IL-6) and interleukin-8 (IL-8) reinforce senescence and modulate the tissue microenvironment through activation of nuclear factor κB (NF-κB) and STAT3 signaling pathways [51–54]. In reality, SASP is far from a static molecular collection but instead exhibits a high degree of context dependence and marked heterogeneity [55].

This heterogeneity is manifested first in SASP polarization: pro-inflammatory SASP (driven by cGAS-STING and NF-κB, enriched in IL-6, IL-8, and chemokines) recruits NK cells and macrophages for early immunosurveillance, whereas TGF-β-enriched SASP suppresses NK and T cell activity, promotes EMT, and builds immunosuppressive stromal niches—diametrically opposite effects from similar molecular triggers [56]. Additionally, the inducing mechanism profoundly influences SASP composition: mitochondrial dysfunction-associated senescence (MiDAS) presents a secretory profile lacking IL-1-dependent components yet enriched in distinctive chemokines such as CCL27 [57]. Cell-type origin specificity constitutes another layer: stromal fibroblasts, epithelial cells, and endothelial cells activate markedly different transcriptional networks upon entering senescence. In CNS tumors such as GBM, the SASP of senescent astrocytes encompasses not only classical pro-inflammatory factors but also specifically secreted glutamate, TGF-β, and specific microRNAs (miRNAs), displaying dual characteristics of prominent neurotoxicity and immunosuppression [51]. Finally, SASP evolution exhibits pronounced temporal dynamics: during the early phase of senescence, “early SASP” driven by IL-1α and NOTCH signaling primarily exerts immune-activating functions [51–54]. However, as senescence persists, sustained epigenetic remodeling and continuous mTOR activation drive a cascade of reprogramming of the secretory profile, irreversibly progressing toward a late-stage composition enriched in TGF-β and immunosuppressive extracellular vesicles (EVs) [54].

Within the complex microenvironment of GBM, this spatiotemporal heterogeneity of SASP, particularly the transition toward a TGF-β-enriched phenotype, serves as a critical bridge linking tumor cells, senescent cells, and immune cells. For instance, when damaged astrocytes enter senescence following chemoradiation, their specific “late SASP” enriched in CXCL1, TGF-β, and selective EVs not only recruits peripheral myeloid cells but also actively “educates” surrounding normal microglia through potent paracrine signaling. This process induces microglial polarization toward the tumor-promoting M2 phenotype and can even promote their intrinsic metabolic exhaustion and immunosenescence [40, 58]. This trans-cellular communication network demonstrates that SASP is the core chemical signal converting static cell cycle arrest into dynamic environmental remodeling. Recent studies have underscored the roles of EVs and exosomes within SASP in promoting tumorigenesis, enhancing cancer cell proliferation, and conferring therapeutic resistance [47, 48]. These vesicles can traverse the blood-brain barrier (BBB) or propagate senescence signals over long distances within the brain parenchyma, endowing neighboring non-cancerous cells with pro-tumorigenic properties. Accurately distinguishing senescent cells and separating them from dormant or drug-tolerant populations remains a critical goal for clinical and translational research [59].

In summary, while the core features and markers of senescence have become increasingly well defined, continued efforts are needed to develop detection methods with greater specificity and sensitivity, as well as targeted clearance strategies for senescence-associated diseases and cancers. Integration of multi-omics approaches spanning transcriptomics, proteomics, epigenomics, and metabolomics holds promise for discovering novel biomarkers and precision targets for senolytic and senomorphic interventions [60]. Such integration could not only identify hotspots of tumor recurrence risk but also reveal senomorphic intervention targets directed against specific secretory factors, such as selective blockade of TGF-β-mediated immunosuppression, thereby precisely disrupting the pro-tumorigenic niche without compromising normal cellular functions. As our understanding of senescence mechanisms deepens, this may lead to transformative clinical therapies.

### Senescence and cancer

**Fig. 2:**
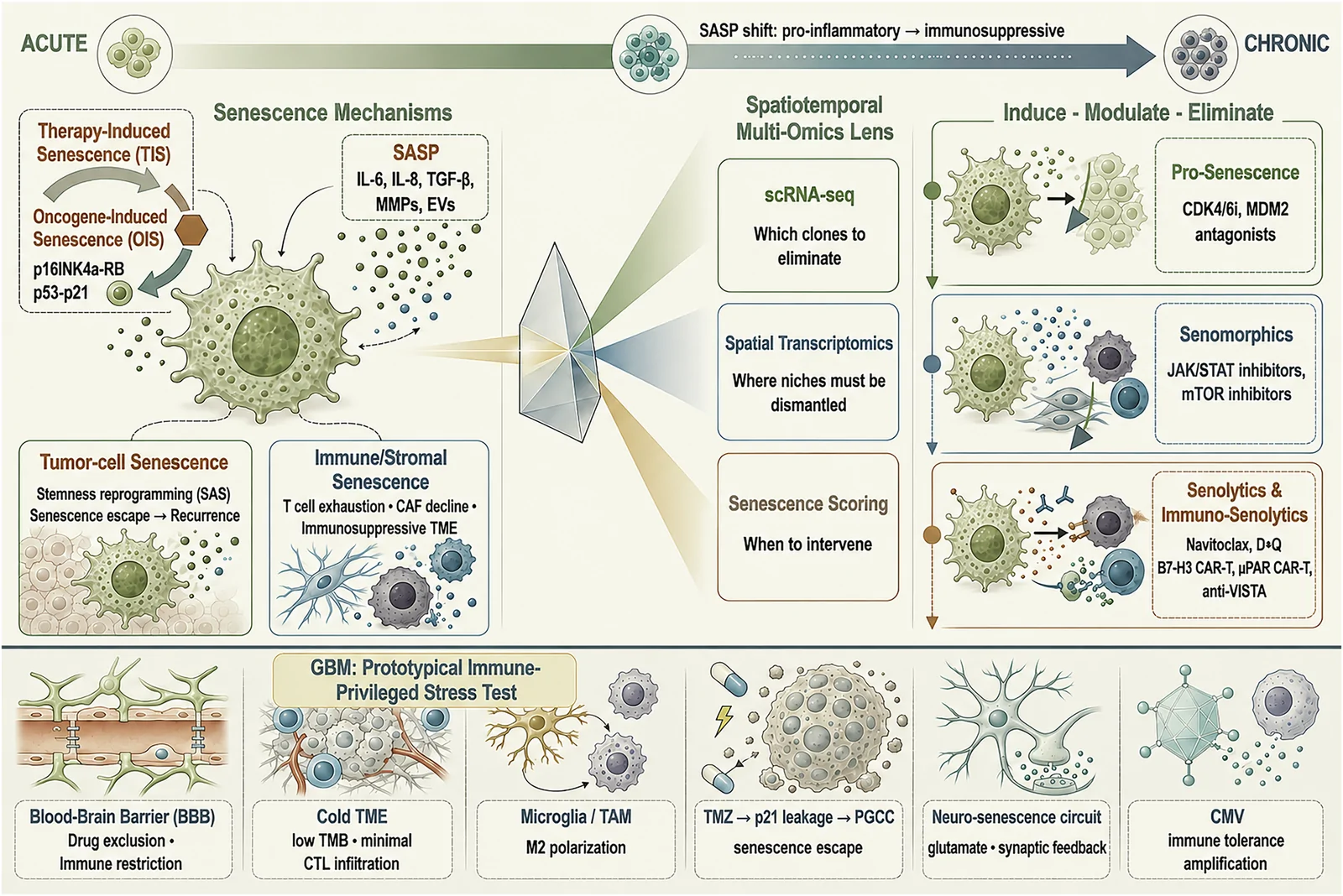
Deciphering and targeting senescence dynamics in glioblastoma (GBM). The upper panel summarizes the transition from acute to chronic senescence, accompanied by a shift in the senescence-associated secretory phenotype (SASP) from pro-inflammatory to immunosuppressive states. Driven by the p16-RB and p53–p21 pathways, senescence shapes both tumor cells and the immune/stromal microenvironment. Spatiotemporal multi-omics, including scRNA-seq, spatial transcriptomics, and senescence scoring, help resolve clonal heterogeneity and niche organization and support a three-step therapeutic strategy: induce senescence, modulate SASP, and eliminate senescent cells. The lower panel highlights GBM as an immune-privileged stress test, emphasizing key barriers to therapy, including the blood-brain barrier, a cold tumor microenvironment, M2-polarized microglia/TAMs, temozolomide-induced polyploid giant cancer cells escaping senescence, neuro-senescence synaptic feedback loops, and CMV-amplified immune tolerance.

Cellular senescence plays a highly complex and spatiotemporally dynamic role in tumorigenesis and progression. Senescence and cancer are intimately linked, but the consequences diverge sharply depending on which compartment becomes senescent. Senescence of tumor cells themselves is initially tumor-suppressive (OIS/TIS-mediated arrest) yet becomes a reservoir of stemness reprogramming and recurrence once cells escape arrest; senescence of stromal and immune cells, by contrast, predominantly reshapes the microenvironment—remodeling the ECM, blunting immunosurveillance, and driving immunosuppression. This compartment-dependent duality, rather than senescence per se, carries a direct therapeutic corollary: an intervention that beneficially clears senescent tumor cells may simultaneously deplete protective senescent immune effectors, so senescence-targeting strategies must be compartment-aware. Initially, senescence functions as a tumor-suppressive mechanism by inducing permanent cell cycle arrest and facilitating immune-mediated clearance of damaged cells, thereby preventing malignant transformation. However, during tissue aging or the progression of established tumors, the accumulation of senescent cells and their pro-inflammatory secretory phenotype remodels the microenvironment and promotes tumor aggressiveness. The net outcome depends on tumor stage and the surrounding microenvironment (Fig. 2).

During early tumorigenesis, senescence in mutated premalignant cells serves as a critical barrier. Cells exposed to oncogenic stresses, such as oncogene activation or DNA damage, undergo irreversible cell cycle arrest, thereby halting the proliferation of damaged cells. This process triggers the release of IL-1α, which initiates SASP, activates NF-κB, and upregulates IL-6 and IL-8, reinforcing growth arrest [61]. These cytokines amplify ROS and maintain the DDR, particularly during OIS. IL-1α also triggers paracrine senescence in neighboring cells, further suppressing tumor progression [62]. In gliomagenesis, this early senescence barrier occurring in neural precursor cells effectively limits the rapid growth of low-grade lesions. However, once these cells acquire mutations that evade the senescence program, such as homozygous deletion of [63]. *CDKN2A*, tumors rapidly progress to highly invasive GBM

When senescent cells are not effectively cleared, particularly when stromal cells within the microenvironment undergo senescence, their SASP generates pro-tumorigenic effects by remodeling the TME and inducing chronic inflammation that supports tumor growth. Key SASP factors, including IL-6, IL-8, and vascular endothelial growth factor (VEGF), promote angiogenesis, EMT, and the maintenance of tumor stemness, thereby facilitating metastasis. Senescent fibroblasts secrete MMPs that degrade the ECM, aiding cancer cell invasion and growth [17]. Furthermore, these NF-κB-dependent SASP factors drive neighboring premalignant cells toward proliferation and invasion, and can reprogram adjacent breast cancer cells into more aggressive phenotypes, fueling treatment resistance [17]. Within the GBM microenvironment, the pro-tumorigenic role of stromal senescence is particularly pronounced. Tissue hypoxia or radiotherapy induces normal astrocytes and endothelial cells into senescence. The SASP secreted by senescent astrocytes not only directly promotes the proliferation and invasion of residual non-senescent glioma cells but can also, through microenvironmental metabolite rearrangement, sustain the survival and self-renewal of glioma stem cells (GSCs), thereby constructing a pro-tumorigenic niche that supports recurrence [64, 65]. IL-6 recruits myeloid-derived suppressor cells (MDSCs), which suppress immunosurveillance by blocking IL-1α signaling and inhibiting the activity of CD8⁺ T cells and NK cells [66]. The effects of SASP depend on tissue context and the senescence trigger; when not completely cleared, SASP fosters a pro-tumorigenic inflammatory environment.

Persistently present senescent cells remodel the TME. SASP-derived MMPs and growth factors promote angiogenesis and tumor cell invasion. Certain SASP factors recruit immunosuppressive elements, facilitating immune evasion. When tumor cells themselves undergo TIS, their fate carries decisive implications for long-term cancer progression. Senescent tumor cells frequently upregulate immune checkpoint molecules such as programmed death-ligand 1 (PD-L1), evading attack by cytotoxic T cells [66]. Mitochondrial DNA released from senescent cells activates the cGAS-STING pathway in MDSCs, leading to enhanced tumor immune evasion [67].

To overcome the proliferative constraints imposed by senescence, tumor cells commonly acquire mutations or inactivate key regulators, thereby escaping cell cycle arrest and developing drug resistance [68]. Moreover, emerging evidence indicates that even tumor cells entering the senescent state have not entirely lost their malignant potential. The epigenetic reprogramming accompanying senescence can induce tumor cells to acquire prominent stem cell-like characteristics, termed senescence-associated stemness (SAS). When these cells ultimately escape senescence, they frequently exhibit greater tumor-initiating capacity and multidrug resistance than the primary tumor, directly driving rapid tumor invasion and recurrence [69].

Although senescent tumor cells cease proliferating, they continuously influence their surroundings through SASP secretion and direct cell-cell interactions, functioning as an “invisible engine” of tumor growth. Their sustained inflammatory and growth signals support the survival and expansion of residual tumor cells. SASP-induced inflammation increases the invasiveness and metastatic potential of non-senescent cancer cells while suppressing antitumor immunity, driving recurrence, and disease progression. Drug-resistant senescent tumor cells represent a major cause of relapse, as they create environments favorable for tumor regeneration. Some senescent cells acquire additional mutations or epigenetic alterations, giving rise to resistant clones. Preclinical studies have demonstrated that administration of senolytics such as ABT-263 (Navitoclax) following chemoradiation significantly delays recurrence and metastasis [67], supporting the rationale for sequential senescence induction followed by senolytic clearance.

## Spatiotemporal multi-omics dissection of cellular senescence

The concept of a “Time-Space Senescence Atlas” refers to an innovative framework that systematically maps and analyzes the emergence, distribution, and evolution of senescent cells in vivo across both temporal and spatial dimensions. Within the complex tumor ecosystem, senescence is neither static nor synchronous but instead represents an intricate dynamic process. Conventional static approaches fail to capture the full complexity of senescence, making atlas construction a pioneering scientific endeavor. By integrating multi-omics technologies, including single-cell transcriptomics, spatial transcriptomics, and epigenomics, with advanced imaging analysis, spatiotemporal atlases can resolve, at high resolution, the emergence patterns, migration trajectories, and heterogeneous evolution of senescent cells in situ (Fig. 2). More importantly, these atlases are profoundly transforming our understanding of TME heterogeneity, providing critical translational coordinates for the precision elimination of drug-resistant senescent clones and the disruption of tumor immunosuppression.

### Constructing the Atlas: from static markers to omics networks

With the rapid advancement of high-throughput sequencing and imaging technologies, senescence atlases have evolved from bulk tissue surveys to cell-level and spatial-level characterization (Table 1). Early studies relied on bulk RNA-seq and microarrays to delineate age-dependent remodeling of immune, metabolic, cell cycle, and DNA repair pathways, but lacked cell-type resolution and spatial context.

**Table 1:** Overview of single-cell and spatial transcriptomic aging studies.

Overview of single-cell and spatial transcriptomic aging studies								
Author	Date	Sample source	Omics	Sequencing platform and technology	Sample size/cell count	Main findings	Accession (GEO / Other)	Reference
Gavish et al.	2023	Pan-cancer human tumors (including GBM)	Single-cell transcriptomics	Various	>1000 tumors	Identified pan-cancer hallmarks of intratumor heterogeneity, including stress-response and senescence-related cellular states.	—	[107]
Troiani et al.	2022	Cancer models treated with senescence-inducing therapies	Single-cell transcriptomics	10x Genomics	~10,000 cells	Identified Mcl-1 as a critical survival factor for therapy-induced senescent (TIS) tumor cells, providing a new senolytic target.	GSE189519	[121]
Tong et al.	2025	Human nasopharyngeal carcinoma (NPC)	Single-cell transcriptomics	10x Genomics	Not specified	Identified senescent epithelial cells and SPP1^+^ macrophages associated with tumor progression and SASP secretion.	—	[122]
Neftel et al.	2019	Human glioblastoma (GBM) patients	Single-cell transcriptomics	Smart-seq2; 10x Genomics	7930 cells	Identified four meta-states in GBM and their plasticity, forming the basis for tracking senescence-like/drug-resistant clones.	GSE131928	[77]
Ravi et al.	2022	Human glioblastoma (GBM) patients	Spatial transcriptomics, scRNA-seq	10x Visium; Smart-seq2	14 patients	Deciphered spatial microdomains of tumor-host interdependence, locating immune-exhausted niches relevant to SASP.	—	[80]
Ela Elyada et al.	2019	Human PDAC patients (*n* = 6) and KPC mice (*n* = 4)	Single-cell transcriptomics	10x Genomics; Illumina HiSeq4000	Human: 21,200 cells; Mouse: 11,260 cells	Defined myCAF/iCAF subpopulations and discovered antigen-presenting CAFs activating CD4⁺ T cells.	GSE129455 (mouse); dbGaP (human)	[76]
Liu et al.	2024	Human glioblastoma (GBM) patients	Spatial transcriptomics, scRNA-seq	10x Visium; 10x Genomics	12 patients	Mapped the perinecrotic niche and spatial segregation of immunosuppressive TAMs, highlighting senescence hotspots in TME.	—	[109]
Jing et al.	2025	Cross-age GBM patients (adult vs. aged)	Single-cell transcriptomics	Multiple	23 GBM patients	Revealed that aging leads to a dramatic elevation of monocyte-derived macrophages (MDMs) in GBM, driving worse prognosis.	GSE235676, GSE135045, GSE103224, GSE148842	[123]

Conventional histochemical and antibody-based approaches have revealed the distribution of senescent cells in tissues: SA-β-gal and lipofuscin staining enable in situ senescent cell localization [70, 71]; immunohistochemistry/immunofluorescence targeting canonical markers (p21^CIP1^, p16^INK4a^, p53, and γH2AX) are constrained by antibody specificity and throughput limitations [72, 73]; high-resolution methods such as ImmunoFISH and super-resolution microscopy (e.g., STORM) further map chromatin alterations at nanoscale [74], although technical complexity limits large-scale application [75] and both remain constrained by fluorophore performance and computational burden.

While these methods have yielded valuable spatial and molecular insights, they lack the scalability required for high-throughput atlas construction. Emerging single-cell transcriptomics and spatial omics approaches are overcoming these limitations, enabling multi-scale charting of senescence trajectories and propelling the field from simple “marker identification” toward systematic “TME network dissection”—laying the methodological foundation for resolving the role of TIS in tumor recurrence.

### Single-cell transcriptomics: deconstructing pathogenic senescent clones in the TME

scRNA-seq provides an unprecedented view of individual cell transcriptomes, enabling the distinction between senescent and non-senescent populations and the discovery of subpopulation heterogeneity within the TME. In tumor research, scRNA-seq has fundamentally overturned the previous stereotype of senescence as a “homogeneous, static endpoint.” Single-cell resolution has revealed that senescence is, in fact, a continuous spectrum of high plasticity.

For instance, in the pancreatic ductal adenocarcinoma microenvironment, scRNA-seq uncovered distinct fibroblast subpopulations within the tumor-associated stroma with bidirectional tumor-regulatory functions, including inflammatory cancer-associated fibroblasts (iCAFs) secreting pro-inflammatory SASP and myofibroblastic cancer-associated fibroblasts (myCAFs) responsible for stromal remodeling [76]. In highly heterogeneous tumors such as GBM, single-cell sequencing has enabled researchers to precisely dissect chemotherapy-induced senescent tumor clones from the complex cellular network and has revealed how these cells upregulate stemness genes and immune evasion receptors through transcriptional reprogramming within the senescent state [77]. The deeper implication is clear: clinical treatment should not indiscriminately pursue widespread senescence induction, as not all senescent cells are tumor-suppressive. scRNA-seq confers the ability to distinguish tumorigenic from protective senescent populations, enabling future therapies to precisely identify and preferentially eliminate “pathogenic senescent clones” with stemness reprogramming potential or strong immunosuppressive phenotypes, thereby avoiding tissue toxicity from indiscriminate targeting.

### Spatial transcriptomics: visualizing senescence–immune spatial niches

While marker-based methods and scRNA-seq capture cell-type diversity, they lose in situ spatial information that is essential for understanding how SASP factors remodel the ECM and neighboring cells to drive tissue remodeling, tumorigenesis, and chronic inflammation [78, 79]. Spatial transcriptomics maps genome-wide expression profiles while preserving tissue architecture, identifying regions with high senescence marker expression and revealing local cellular composition.

The true breakthrough of spatial transcriptomics lies in its physical-dimension confirmation that paracrine senescence and immunosuppressive gradients genuinely exist in vivo. In GBM tumor models, spatial transcriptomics has not only identified isolated senescent cells but has also precisely localized senescence “microdomains” forming around pseudopalisading necrotic zones. Studies have confirmed that these hotspots, composed of senescent stromal cells and TIS tumor cells, release high local concentrations of SASP factors such as TGF-β, and are spatially and physically superimposed with exhausted T cells and M2-polarized macrophages [80]. This finding provokes a profound reconsideration of therapeutic strategy: the hazard of senescence lies less in the persistence of individual cells than in the pro-tumorigenic spatial niche they construct. By enabling the precise localization of key cellular subsets and microenvironmental alterations, spatial transcriptomics offers an entirely new perspective for future antitumor interventions. Through identification of senescence hotspots and enriched signaling niches, therapeutic targets extend beyond mere senescent cell clearance to encompass the complete dismantlement of the immunosuppressive spatial barriers through localized delivery strategies, such as nanoparticle-based drug systems that disrupt SASP concentration gradients.

### Innovations in spatial proteomics

Because mRNA abundance does not reliably predict protein level or activity, mass spectrometry-based and high-parameter antibody-based spatial proteomic platforms complement transcriptomic atlases, resolving subcellular remodeling, post-translational modifications, and the spatial localization of rare senescent populations through simultaneous multiplexed protein detection [75]. Critically, spatial proteomics bridges the “gene-to-antigen” gap left by transcriptomics—where post-transcriptional regulation and cell-surface vesicle shedding decouple candidate markers from druggable targets. By verifying in situ the surface co-expression of classical markers alongside novel targets such as uPAR and senescence-associated checkpoints B7-H3 and VISTA (discussed below) within intact tissue architecture, it provides reliable “navigation maps” for designing multispecific antibodies, antibody-drug conjugates (ADCs), and logic-gated CAR-T cells, reducing off-target toxicity and accelerating precision senolytic immunotherapy.

### Clinical translation: from scoring systems to precision interventions

The development of spatiotemporal atlases of cellular senescence marks a significant advance in understanding the dynamic behavior of senescent cells. These atlases chart the distribution, evolution, and fate of senescent cells across temporal and spatial dimensions, providing a panoramic view of their roles in diverse tissue and disease contexts. More critically, drawing on the accumulation of massive single-cell and spatial datasets, researchers have developed a series of bioinformatics tools and scoring systems with direct clinical applicability.

Drawing on accumulating single-cell and spatial datasets, bioinformatics tools and scoring systems have been developed with direct clinical applicability. Transcriptome-based scores quantify senescence burden and stratify prognosis—exemplified by Senescore in bladder cancer (guiding molecular subtyping) [81] and the Senescence Risk Relevant Score (SRRS) in colorectal cancer, where high-risk patients show poorer prognosis, greater immune infiltration, and reduced stemness [82]. Cross-species and cell-type-specific gene sets such as SenMayo [59] and SenePy [72] then improved fidelity to in vivo senescence states beyond in vitro-derived signatures.

Most groundbreaking is the advent of single-cell classifiers such as SenCID, which assign senescent cells to distinct molecular identities and predict differential sensitivity among subpopulations to specific senolytics such as Navitoclax [73]—marking the transition from mechanistic description toward guiding precision senescence intervention. Collectively, these tools convert descriptive atlases into actionable therapeutic coordinates: single-cell classifiers nominate which senescent subpopulations to eliminate, spatial maps reveal where immunosuppressive niches must be dismantled, and longitudinal scoring defines when to intervene—directly informing the senescence-targeted strategies and the GBM application developed in the following sections.

## Senescence-immunity interactions in the tumor microenvironment

During tumorigenesis and progression, the immune system serves not only as the frontline defense against malignant cell expansion but also as a central force shaping TME heterogeneity [83]. Classical tumor immunology holds that tumors continuously edit and reprogram infiltrating immune cells, achieving the transition from immunosurveillance to immune evasion. However, when the variable of cellular senescence is introduced into the TME, the complexity of the immune landscape is amplified exponentially. Under the dual context of tumor burden and senescence, the local immune system is characterized by pronounced state bipolarization and spatiotemporal dependence: acute senescence induced by chemotherapy or oncogenic stress typically exhibits high immunogenicity, functioning as a potent endogenous adjuvant that activates both innate and adaptive immunity, thereby transiently reshaping a highly activated antitumor microenvironment [84]. Conversely, prolonged tumor burden and chronic senescence signals, particularly persistent SASP, pathologically converge to induce profound metabolic exhaustion, phenotypic aging (immunosenescence), and the formation of dense immunosuppressive networks among TME immune cells, ultimately leading to comprehensive collapse of antitumor immunity [51]. Therefore, a thorough dissection of how cellular senescence reshapes immune characteristics within the TME is not only at the theoretical frontier of understanding drug resistance and tumor recurrence but also constitutes a key prerequisite for developing next-generation tumor immunotherapies, such as the combined use of senolytics with immune checkpoint blockade [85]. Critically, the senescence program operates in two distinguishable compartments here: tumor cells co-opt senescence-associated stemness and checkpoint upregulation to evade clearance, whereas infiltrating immune cells are driven into a bystander senescence and exhaustion that disarms surveillance—two processes that share molecular machinery (p16^INK4a^/p21, DDR) but demand opposite therapeutic responses: eliminate the former, rejuvenate the latter.

### Early immune clearance and the senescence–immunity–tumor tripartite network

In young, healthy tissues and during early tumorigenesis, senescent cells are rapidly recognized and eliminated by the immune system [86]. This process depends on highly coordinated chemotactic recruitment and receptor-ligand recognition pathways. Senescent cells release CCL2 and CXCL8 through their SASP, which bind to CCR2 on monocytes/macrophages and CXCR2 on neutrophils, respectively, driving directed migration of innate immune cells toward senescent lesions [87]. During the effector recognition phase, NK cells detect stress-induced surface ligands such as MICA/B and the ULBP family through the NKG2D receptor, subsequently activating downstream DAP10 or DAP12 adapter protein signaling cascades, and eliminating senescent cells through perforin- and granzyme-mediated cytotoxicity [88]. Macrophages, assisted by natural IgM antibodies, phagocytose senescent fibroblasts by recognizing oxidized phospholipids exposed on senescent cell surfaces or by detecting downregulated CD47 signaling. Invariant natural killer T (iNKT) cells also contribute to tumor senescence surveillance via CD1d recognition on senescent cells [89]. Adaptive immunity is likewise engaged: CD8⁺ T cells target senescent cells presenting aberrant antigens, including endogenous retroviruses or viral peptides [85]. NK cells, macrophages, neutrophils, CD4⁺/CD8⁺ T cells, and iNKT cells cooperate synergistically to maintain tissue homeostasis and prevent senescent cell accumulation.

**Fig. 3:**
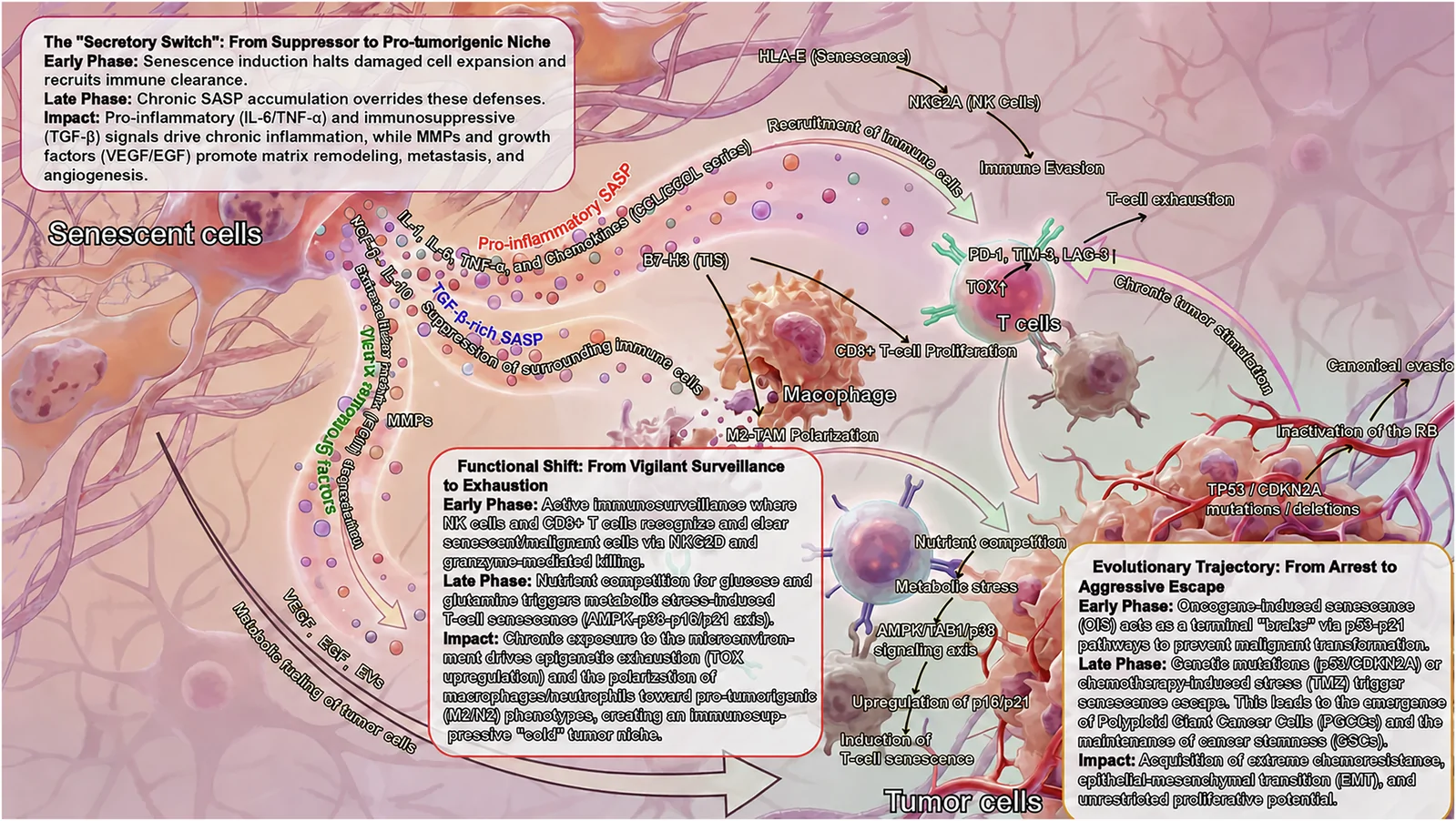
Molecular mechanisms of the senescence-immunity-tumor tripartite network. This figure illustrates the interactive logic within the TME driving malignant progression. Senescent cells execute a secretory switch, where chronic accumulation leads to persistent release of SASP (IL-1, IL-6, TNF-α, and TGF-β) and MMPs, driving inflammation, immunosuppression, and ECM remodeling, while VEGF, EGF, and EVs provide metabolic fueling. Immune cells undergo a functional shift triggered by nutrient competition and metabolic stress, activating the AMPK/TAB1/p38 axis and ATM-mediated DDR, resulting in T-cell senescence (p16/p21 upregulation). Chronic stimulation further drives TOX and inhibitory receptor (PD-1, TIM-3, and LAG-3) expression, alongside novel checkpoints (B7-H3 and HLA-E/NKG2A) that facilitate M2-TAM polarization and evasion. Tumor cells utilize diverse escape programs: .*TP53*/*CDKN2A* deficiency leads to RB inactivation and cell cycle reentry, while TMZ-induced stress triggers p21 leakage and aberrant DNA re-replication, forming chemoresistant PGCCs and maintaining GSC stemness.

At this pathological convergence point, cellular senescence, the immune system, and the tumor establish a highly dynamic senescence–immunity–tumor tripartite regulatory network. Within this network, oncogenic stress drives cells into senescence; senescent cells actively remodel the local immune microenvironment through SASP factor secretion; and the efficacy of immunosurveillance within the microenvironment reciprocally determines the ultimate fate of senescent cells. When immune function is intact, the senescence network serves as a barrier blocking tumorigenesis. However, when the rate of senescent cell generation exceeds immune clearance capacity, uncleared senescent clones become microenvironmental hubs driving tumor progression (Fig. 3).

### Immune evasion mechanisms and novel checkpoints

When immune clearance capacity within the TME declines, senescent cells that are not promptly eliminated undergo adaptive evolution, constructing a robust immunosuppressive shield. Initially, NK cells identify and clear senescent targets through NKG2D recognition, but prolonged SASP exposure, particularly to IL-6, downregulates activating receptors while upregulating inhibitory receptors, including NKG2A and PD-1. Senescent cells commonly express HLA-E, which binds NKG2A to suppress NK cytotoxicity, or overexpress ganglioside GD3, which engages Siglec-7 on NK cells to block activation and facilitate evasion [90]. Furthermore, highly expressed p16^INK4a^ in senescent cells stabilizes cell-surface PD-L1 expression by inhibiting CDK4/6-mediated ubiquitination and degradation pathways, thereby profoundly suppressing T cell function [6]. Macrophages are initially polarized toward the M1 phenotype by SASP to assist clearance; however, prolonged exposure drives their conversion to the M2 phenotype, producing IL-10 and TGF-β and fostering a tolerogenic environment.

In response to the limitations of PD-1/PD-L1-targeted immunotherapy in certain solid tumors, recent studies have revealed that senescent cells can also achieve multilayered immune evasion through upregulation of novel immune checkpoints. In GBM, TIS tumor cells and their secreted EVs significantly upregulate B7-H3 (CD276) expression. B7-H3 not only directly blunts the proliferation and cytotoxic activity of infiltrating CD8⁺ T cells but also promotes macrophage polarization toward a pro-tumorigenic phenotype within the microenvironment [91]. Moreover, MDSCs interacting with senescent cells have been found to express high levels of V-domain Ig suppressor of T cell activation (VISTA), a key inhibitory factor maintaining immune cell quiescence that profoundly suppresses T cell activation [92]. Concurrently, the poliovirus receptor (PVR/CD155) upregulated on senescent cell surfaces engages in high-affinity crosslinking with TIGIT receptors on NK cells and T cells, inducing deep exhaustion of effector lymphocytes [93]. These novel checkpoints, together with classical pathways, constitute a dense immunosuppressive network that renders senescent tumor cells exceedingly difficult to eradicate.

### Immune cell exhaustion and GBM-specific bidirectional regulation

Within this tripartite network, regulatory effects are highly bidirectional. The TME does not merely passively endure immune attack; the senescence signals within it, such as metabolic stress, can actively induce intrinsic senescence and functional exhaustion in infiltrating immune cells. Metabolic competition represents a core driver of local immunosenescence within tumors. For instance, tumor-infiltrating Tregs deplete local glucose, depriving effector T cells of their energy substrates. This metabolic stress triggers ATM-mediated DDR, upregulating p21^CIP1^, p16^INK4a^, and p53 through the AMPK–TAB1–p38 MAPK signaling cascade, directly inducing cell cycle arrest and intrinsic senescence in effector T cells [94, 95]. NK cells exposed to the aberrant tumor microenvironment similarly undergo accelerated decline, manifesting as diminished cytotoxicity and increased KLRG1 expression, which attenuates their capacity to eliminate malignant or senescent cells [96].

Within the unique BBB and CNS ecosystem of GBM, this tripartite network exhibits pronounced tissue specificity. Therapy-induced senescence in CNS-resident cells, particularly astrocytes, establishes a highly specialized paracrine loop that directly blunts local immunosurveillance. Rather than executing their phagocytic duties, resident microglia are actively reprogrammed by these senescence signals into an exhausted, tumor-protective state [40, 64]. This profound neuro-immune rewiring exemplifies how localized senescence can completely subvert innate immune defenses within highly immune-privileged niches.

### Coevolution of senescence, immunity, and tumors: from defense to promotion

Tumor initiation, progression, and treatment response form a dynamically evolving process in which senescence, immunity, and the TME interact within a finely tuned network. During early tumorigenesis, senescent tumor cells, immune effectors, and transformed cells create a positive feedback loop: senescent tumor cells secrete inflammatory SASP factors such as IL-1α, IL-6, and TNF-α, which reinforce their own arrested state while simultaneously recruiting and activating NK cells and CD8⁺ T cells through chemokines including CCL2 and CCL5. These cytotoxic lymphocytes eliminate premalignant lesions through perforin/granzyme mechanisms and Th1-mediated IFN-γ responses. Dendritic cells process and present senescence-associated antigens to T cells, strengthening adaptive immune memory and consolidating this defensive mechanism [97]. Notably, this regulatory network is not unidirectionally immunosuppressive; under specific conditions, the activated immune system can likewise actively drive tumor cells into senescence. For instance, IFN-γ and TNF-α secreted by effector Th1 cells can potently drive tumor cells that have escaped apoptotic pathways into permanent senescence arrest, thereby confining tumor lesions to a long-term dormant phase [98].

When senescent cells fail to be cleared promptly and persist, their SASP, including VEGF, MMP-2/9, and TGF-β, remodels the ECM and vasculature to support tumor growth and invasion. Chemotactic signals such as CCL2 and CXCL8 recruit and polarize Tregs and MDSCs, which secrete IL-10 and TGF-β to suppress NK and CD8⁺ T cell function. The GD3–Siglec-7 and HLA-E–NKG2A axes, together with the aforementioned novel immune checkpoints, further suppress NK- and T cell-mediated clearance, forming a “tripartite pro-tumorigenic network” involving senescent cells, immunosuppressive leukocytes, and cancer cells [99, 100]. Nutrient competition between tumor cells and senescent cells for glucose and glutamine induces metabolic stress in effector T cells, upregulating PD-1 and NKG2A, thereby exacerbating immunosenescence. Simultaneously, SASP triggers epigenetic remodeling, such as STAT3-mediated activation of the PD-L1 enhancer, maintaining immune checkpoint expression on both tumor and senescent cells. This “metabolic–epigenetic” barrier impedes immune clearance [101].

Given these dual-edged interactions, monotherapies targeting either immune checkpoints or senolytic clearance typically confer only transient benefit. Recent studies have proposed combining JAK/STAT or BRD4 inhibitors with immune checkpoint blockade and senolytics to reduce deleterious SASP, reprogram metabolism, and reinvigorate exhausted immune cells, thereby extending tumor control and preventing relapse [84]. Future strategies should prioritize real-time monitoring of SASP profiles and immune phenotypes within the TME to dynamically tailor combinatorial therapies and break the pro-tumorigenic senescence–immune cycle.

The dual role of senescence and immunity exhibits striking spatiotemporal heterogeneity across different tumor stages: early cooperation forms an antitumor barrier, whereas late-stage cooperation drives immune evasion and tumor progression. Disrupting this balance presents therapeutic challenges yet also offers opportunities for precision intervention. Senolytic therapies targeting accumulated senescent cells, combined with senomorphic SASP reprogramming, can restore immune competence and halt pro-tumorigenic processes. Simultaneously, strategies to delay immunosenescence or reverse immune exhaustion will enhance clearance capacity, proving critical for next-generation immuno-oncology therapeutics [41].

## Senescence-targeted cancer therapeutic strategies

**Fig. 4:**
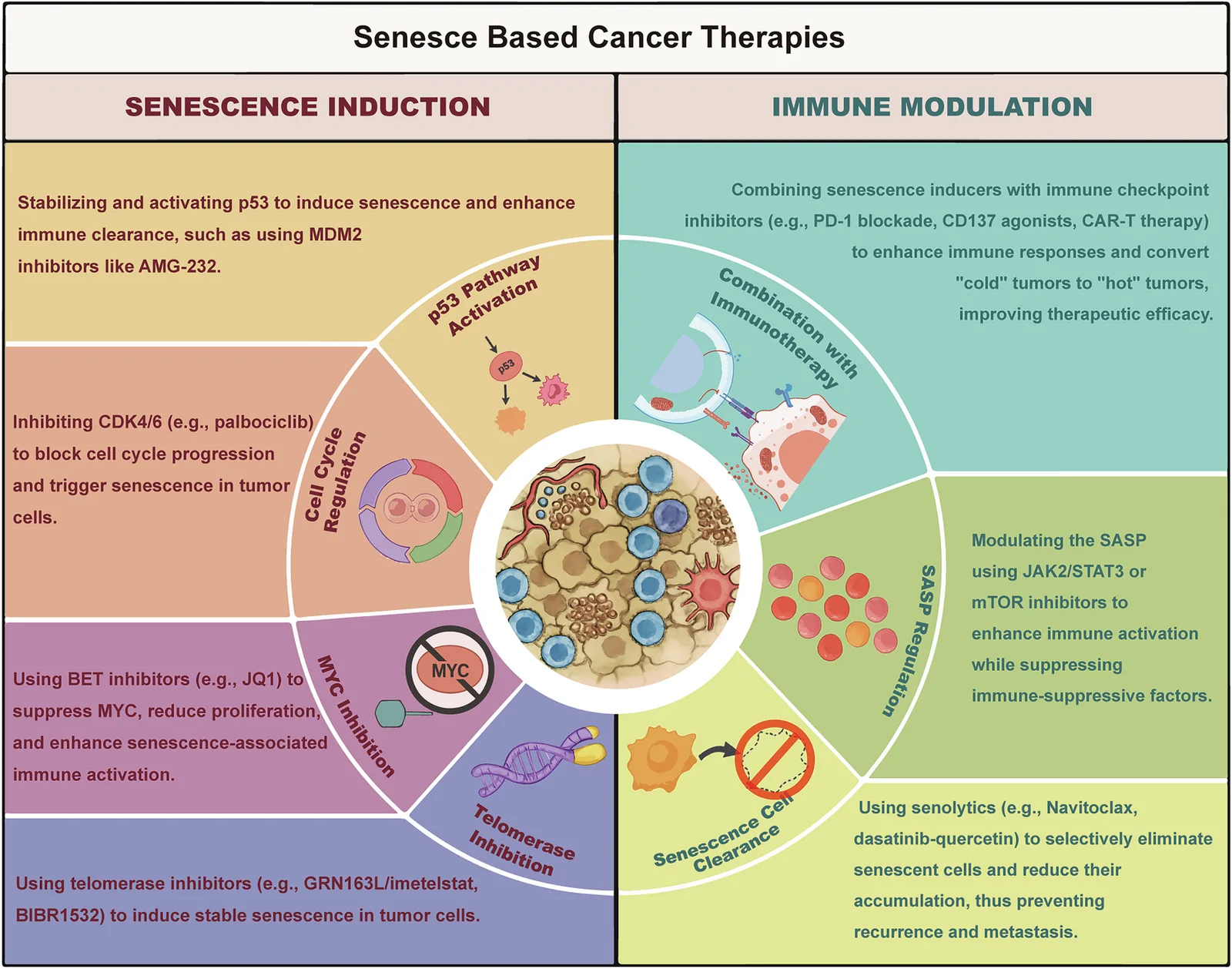
Senescence-based cancer therapies. Therapeutic strategies targeting cellular senescence in cancer include telomerase inhibitors (e.g., GRN163L), CDK4/6 inhibitors (e.g., palbociclib), MDM2 inhibitors (e.g., RG7112), BET inhibitors (e.g., JQ1), JAK2/STAT3 and mTOR inhibitors (e.g., rapamycin), and senolytics (e.g., Navitoclax). These therapies induce senescence, enhance immune responses (e.g., SASP), and reduce relapse risk. Combining senescence inducers with immunotherapies (e.g., PD-1 inhibitors) can improve immune surveillance and long-term tumor control.

Cellular senescence, as a critical barrier preventing malignant transformation, has inspired “pro-senescence” therapies. These therapies selectively induce tumor cells into stable growth arrest, halting proliferation and activating immunosurveillance to provide durable antitumor responses, a fundamental departure from conventional cytotoxic chemotherapy (Fig. 4). However, given the complex dynamics of senescent cells within the microenvironment, modern senescence-based therapies have evolved into a “one-two punch” strategy: first inducing senescence or blocking senescence escape, then deploying senomorphics to neutralize harmful secretions, and finally using senolytics or novel immunotherapies to precisely eliminate senescent clones.

### Pro-senescence and prevention of senescence escape

In the treatment of most solid tumors, cyclin-dependent kinase 4/6 (CDK4/6) inhibitors, including palbociclib and ribociclib, represent key targets for senescence induction. They arrest tumor cells in G₁ phase, triggering autonomous SASP to recruit NK cells and macrophages [102]. Concurrently, MDM2 antagonists of the Nutlin class (RG7112, AMG-232) that activate the p53 pathway stabilize p53, enforce durable senescence, and downregulate pro-tumorigenic SASP factors such as IL-6 [103]. Additionally, telomerase inhibitors, such as imetelstat, promote senescence through accelerated telomere attrition [8].

However, in GBM, the senescence barrier is heavily influenced by *IDH* mutation status. In *IDH*-mutant gliomas, the oncometabolite 2-hydroxyglutarate (2-HG) produced by the mutant enzyme profoundly inhibits α-ketoglutarate-dependent demethylases, resulting in genome-wide DNA and histone hypermethylation (the CpG island methylator phenotype, CIMP). This epigenetic silencing directly “locks” key senescence regulatory genes, particularly *CDKN2A*. *IDH* mutation inhibitors, such as ivosidenib or DNA methyltransferase inhibitors for epigenetic remodeling, can release hypermethylation-mediated repression and reawaken the tumor cell senescence program, a critical mechanism for delaying progression from low-grade to high-grade disease. Once *IDH*-mutant astrocytomas undergo homozygous deletion of *CDKN2A/B*, tumors irreversibly lose the senescence barrier and directly progress to WHO grade 4.

Conversely, in *IDH*-wildtype GBM, TMZ and radiotherapy constitute the principal foundation for inducing TIS. However, as noted earlier, TMZ-induced senescence is often incomplete, and tumor cells are highly susceptible to genomic re-replication through the p21 leakage mechanism, forming polyploid giant cells that ultimately escape senescence. Therefore, co-administration of CDK4/6 inhibitors or Aurora kinase A (AURKA) inhibitors to stabilize cell cycle arrest represents an important adjunctive measure to prevent senescence escape following TMZ treatment

### Senomorphics for microenvironmental remodeling

SASP possesses dual tumor-promoting and tumor-suppressing properties. Pharmacological interventions such as JAK2/STAT3 inhibitors or mTOR inhibitors, including rapamycin, can function as senomorphic agents, fine-tuning SASP to suppress immunosuppressive factors while preserving its immune-stimulatory components.

In GBM treatment, SASP modulation carries decisive clinical significance. The stromal and tumor senescence induced by TMZ and radiotherapy generates a GBM-specific SASP that not only provides nutritional support for GSCs but also profoundly reshapes the microglia–tumor-associated macrophage (TAM) interactive network, driving it toward a highly immunosuppressive M2 polarization state. Through application of JAK/STAT inhibitors such as ruxolitinib, clinicians can specifically deprive senescent cells of their capacity to secrete TGF-β and IL-6 without directly killing cells. This effectively severs the chemical communication through which senescent cells “educate” microglia, thereby reversing the immunosuppressive tension within the microenvironment and reducing the risk of recurrence [101].

### Senolytics and targeted immune clearance

As the “eliminate” arm of the induce–modulate–eliminate framework, senolytics convert transient senescence induction into durable tumor control. While senescence induction initially impedes tumor growth, the sustained accumulation of residual senescent cells drives metastasis and recurrence. Senolytics, such as the BCL-2 family inhibitor Navitoclax and the dasatinib plus quercetin (D + Q) combination, selectively induce apoptosis in senescent cells. In the clinical scenario of GBM, thorough clearance of senescent cells holds dual strategic significance: eliminating drug-resistant tumor clones and, equally importantly, mitigating neurological “collateral damage.” Pro-senescence therapies, particularly whole-brain radiotherapy, inevitably induce normal astrocytes and neurons into senescence. The senescence of these normal neural stromal cells and their chronic inflammatory secretions are directly responsible for white matter injury, chronic neuroinflammation, and the severe post-treatment cognitive dysfunction experienced by patients, commonly referred to as “chemo-brain.” Clearance of these damaged stromal cells with senolytics such as Navitoclax has been demonstrated to effectively reverse neurotoxicity and improve long-term quality of life for patients [64].

However, conventional senolytic small molecules face severe constraints in CNS tumor applications due to limited BBB permeability and systemic toxicity. Accordingly, “immuno-senolytic” strategies developed on the basis of the novel immune checkpoints described above are demonstrating revolutionary promise. Since pathogenic senescent cells, particularly senescent GBM cells, selectively overexpress B7-H3 (CD276) and VISTA on their surfaces, these molecules become ideal specific antigen targets. Currently, B7-H3-engineered CAR-T cell therapy and blocking antibodies designed to reverse VISTA-mediated quiescence are being deployed for precise identification and targeted elimination of senescent clones within the microenvironment [91]. Combined with CAR-T cells targeting uPAR upregulated on senescent cells [104], these integrated approaches not only circumvent nonspecific toxicity but also deeply reconstruct the TME, establishing durable immunosurveillance and sustained tumor control.

Despite the broad promise of these strategies, challenges persist in clinical translation, including senescent cell heterogeneity, drug delivery barriers imposed by the BBB, and potential on-target off-tumor toxicity. Future research should elucidate the dynamic balance between senescence and immunity within the TME, determine optimal therapeutic windows, and develop safe formulations based on nano-delivery technologies or checkpoint-specific targeting. Understanding the role of senescence in immune evasion is critical for advancing pro-senescence therapies into the era of precision oncology.

## The senescence–immune axis in glioblastoma

Having established the pan-cancer senescence–immunity framework, the multi-omics tools that resolve it, and the induce–modulate–eliminate therapeutic logic, we now integrate these threads in glioblastoma—a setting that does not merely exemplify but actively stress-tests every element of the framework. GBM, the most aggressive primary brain tumor, represents the ultimate clinical challenge for senescence-immune-targeted therapies. Despite maximal safe resection, radiotherapy, and TMZ, median survival remains 12–15 months. While GBM shares core senescence pathways and immune evasion mechanisms with other solid tumors, its residence within the immune-privileged CNS introduces distinctive features that fundamentally alter the behavior and therapeutic tractability of the senescence–immunity–tumor tripartite network.

The BBB restricts both drug penetration and immune cell trafficking, creating a “cold” immune landscape with minimal cytotoxic T-cell infiltration [105]. The GBM TME is dominated by CNS-resident cells: microglia constitute 30–50% of the tumor mass, alongside astrocytes, oligodendrocytes, and neurons [106]. scRNA-seq profiling has revealed enrichment of neural progenitor-like, mesenchymal-like, and astrocyte-like tumor cell states, contrasting with epithelial-driven programs of extracranial malignancies [77, 107]. GBM exhibits characteristically low TMB and neoantigen load, largely accounting for the failure of single-agent immune checkpoint blockade [108]. Spatial transcriptomics has revealed GBM-specific “senescence hotspots” where senescent cells interact with TAMs and hypoxic stroma, amplifying chronic inflammation and invasion [109].

Beyond these microenvironmental distinctions, GBM senescence possesses unique molecular features. Senescent GBM cells undergo distinctive metabolic reprogramming, shifting toward glycolysis and fatty acid oxidation to survive within the nutrient-limited, BBB-restricted milieu—diverging from the oxidative phosphorylation-dominant phenotype of peripheral senescent cells, with direct implications for metabolically targeted senolytics [110]. Additionally, irradiated GBM cells release ATP and undergo pronounced P2X7 purinergic receptor isoform switching, downregulating P2X7A while upregulating the pro-growth variant P2X7B—constituting a core driver of radiotherapy resistance and senescence-mediated microenvironmental remodeling [111].

Two GBM-specific senescence behaviors deserve emphasis because they convert standard-of-care therapy into a driver of relapse. First, TMZ-induced senescence in GBM is characteristically incomplete: a subset of glioma cells exhibits “p21 leakage,” bypassing durable arrest to undergo genomic re-replication into polyploid giant cancer cells that re-enter the cell cycle as chemoresistant, stemness-high progeny—an escape route largely absent in TMZ-naïve peripheral tumors [49]. Second, the GBM-specific SASP is neuro-adapted: senescent astrocytes and TIS tumor cells secrete a CXCL1/TGF-β/glutamate-enriched, EV-laden profile that not only sustains glioma stem cells but actively educates resident microglia and tumor-associated macrophages (TAMs) toward irreversible M2 polarization and their own inflammatory senescence—manufacturing the immune-desert phenotype that distinguishes GBM from peripheral senescence niches and rendering the TME refractory to both immunotherapy and senolytics without targeted disruption of this microglia–TAM axis.

The immunological consequences of senescence in GBM are equally distinctive. Radiation-induced astrocyte senescence generates a TGF-β- and CXCL1-enriched SASP that drives microglia toward irreversible M2 polarization and inflammatory senescence, creating an “immune desert” far more refractory than peripheral tumors [112]. GBM harbors a self-reinforcing neuro-senescence circuit: senescent cells release excitotoxic glutamate that stimulates neurons, which in turn provide synaptic feedback, suppressing microglial activation [113]. Furthermore, CMV infection amplifies immunosuppression through a GBM-specific mechanism: senescent cells present CMV antigens to exhausted CD4^+^ T cells, promoting tolerance rather than cytotoxicity, while CMV-driven expansion of terminally differentiated memory T cells and M2 macrophage polarization creates a virally mediated immunosuppressive amplification loop [114].

These unique features necessitate GBM-tailored therapeutic strategies. Clearing radiation-induced senescent astrocytes reverses immunosuppression and enhances radiotherapy efficacy through disruption of the TNF-α–Myc–Max–CXCL1 axis [40]. Combining TIS with senolytics such as Navitoclax or SMAC mimetic Birinapant enables selective clearance while sparing normal brain tissue [39, 115]. To counter the immunosuppressive TME, emerging CNS-specific strategies include nanoparticle-delivered senolytics that bypass the BBB, glutamate antagonists disrupting the neuro-senescence circuit, CSF-1R inhibitors such as PLX3397 depleting immunosuppressive microglia, targeted P2X7B blockade disrupting purinergic pro-tumorigenic signaling [111], and anti-CMV therapies including valganciclovir reversing virus-mediated immune tolerance [116]. These integrated approaches targeting the senescence–immune axis represent critical pathways toward improving outcomes in this uniformly fatal disease.

## Discussion and perspectives

Over the past decade, tumor biology has undergone a profound paradigm shift: cellular senescence is no longer viewed merely as a passive cell cycle endpoint resulting from telomere attrition or DNA damage but has been redefined as a highly active and remarkably plastic ecological architect of the TME [83]. As this review has systematically elucidated, senescence is neither purely tumor-suppressive nor purely tumor-promoting; rather, it occupies the core of a senescence–immunity–tumor tripartite network, within which senescent cells remodel the surrounding stroma and immune compartment through their spatiotemporally dynamic SASP [50], while the immune system oscillates between immunosurveillance and exhaustion, ultimately determining whether senescent clones are cleared or become covert origins of drug resistance and recurrence [6, 84].

The complexity of this tripartite interaction reaches its zenith within the CNS, which possesses strong immune-privileged properties, making GBM the ultimate clinical challenge for testing senescence–immune-targeted therapies. Compared with peripheral solid tumors, GBM confronts both physical and biological barriers: the BBB blocks effective infiltration by the vast majority of macromolecular drugs and immune cells; the extremely low TMB confers a natural “cold tumor” resistance to conventional single-agent immune checkpoint blockade [108]. More critically, the unique neuro-glial interactions of the CNS amplify and distort senescence effects. For instance, aberrant metabolites released by senescent cells, such as glutamate, not only directly alter microenvironmental metabolism but also induce pathological neuronal excitation, which, through synaptic feedback circuits, drives irreversible M2 polarization and inflammatory senescence of brain-resident microglia [112, 113]. This macroscopic pro-tumorigenic ecosystem, which transcends conventional immunology, renders the immunosuppressive network in GBM extraordinarily resilient.

Building upon this understanding, we must critically reassess current standard-of-care treatments. The GBM standard therapy, centered on surgery, radiotherapy, and TMZ (the Stupp regimen), while effectively killing large numbers of rapidly proliferating tumor cells, has, in fact, fallen into a severe clinical paradox. These cytotoxic therapies inevitably drive residual tumor cells, normal astrocytes, and even microvascular endothelial cells into TIS [40, 58]. In essence, conventional chemoradiation inadvertently activates a vicious cycle within the senescence–immunity–tumor axis: senescent cells that cannot be cleared by the exhausted immune system not only acquire enhanced invasiveness and multidrug resistance through SAS reprogramming [69] but also provide the ideal microenvironmental sanctuary for GSC enrichment [39]. Moreover, the widespread senescence of normal neural stromal cells constitutes an important pathological basis for the severe post-treatment cognitive dysfunction experienced by patients [66]. If not supplemented with senescent cell clearance, cytotoxic killing alone will struggle to break the inexorable cycle of tumor recurrence. These considerations underscore the emerging consensus that the quest to define and therapeutically target cellular senescence in cancer must move beyond simple induction toward integrated, context-aware intervention [58].

Looking forward, breaking this clinical impasse requires a multidimensional spatiotemporal intervention philosophy that transcends single-target approaches. First, at the level of precision microenvironmental analysis, single-cell and spatial multi-omics technologies must be more broadly introduced into preclinical evaluation to finely delineate the spatial coordinates of senescence–immune microdomains, enabling intervention at the optimal therapeutic window prior to tumor recurrence [73]. Second, regarding systematic iteration of therapeutic strategies, the simple “pro-senescence” approach must be upgraded to the synergistic “induce–modulate–eliminate” combination, as articulated in the evolving framework for senescence-targeted cancer therapy [117]. To address the physical barrier of the BBB, developing biomimetic nanoparticle-based targeted delivery of senolytic small molecules represents a frontier direction for reducing systemic toxicity and achieving precision clearance within the brain [116].

Most compellingly, as novel immune checkpoints on senescent cell surfaces, including B7-H3 and VISTA, continue to be deciphered, a new pathway deeply integrating senolytics with immunotherapy, termed immuno-senolytics, has been established [91]. In the future, the use of CAR-T cells targeting senescence-specific antigens [104], bispecific antibodies, or the combination of oncolytic viruses [118] with macrophage-reprogramming agents such as CSF-1R inhibitors to “heat” immunologically cold lesions holds promise for completely dismantling the immune barriers erected by senescent cells. Furthermore, given the role of latent viral infections such as CMV in amplifying CNS senescence and macrophage polarization phenotypes, targeted antiviral interventions may constitute a potential synergistic strategy for breaking immune tolerance in GBM [114, 119]. Additionally, the contribution of non-cellular TME components such as intratumoral microbiota to senescence-associated immune remodeling [120] opens an entirely new dimension of investigation, suggesting that comprehensive TME reconditioning may require addressing both cellular and microbial determinants of immunosuppression.

In conclusion, the future of precision oncology should not be confined to continuously dividing cancer cells but must also commit to remodeling the microenvironment to thoroughly destroy the ecological soil upon which cancer cells depend. Through exploration of these highly integrated senescence-targeted intervention regimens in adaptive clinical trials, we stand to fundamentally disrupt the vicious senescence–immunity–tumor cycle, bringing durable survival benefits and the ultimate possibility of cure to patients with GBM and other refractory solid tumors.

## References

[1] Campisi J, d’Adda di Fagagna F. Cellular senescence: when bad things happen to good cells. Nat Rev Mol Cell Biol. 2007;8:729–40. 10.1038/nrm2233 17667954

[2] Childs BG, Baker DJ, Kirkland JL, Campisi J, van Deursen JM. Senescence and apoptosis: dueling or complementary cell fates? EMBO Rep. 2014;15:1139–53. 10.15252/embr.201439245 PMC4253488 25312810

[3] Pribluda A, Elyada E, Wiener Z, Hamza H, Goldstein RE, Biton M, et al. A senescence-inflammatory switch from cancer-inhibitory to cancer-promoting mechanism. Cancer Cell. 2013;24:242–56. 10.1016/j.ccr.2013.06.005 23890787

[4] Sieben CJ, Sturmlechner I, van de Sluis B, van Deursen JM. Two-step senescence-focused cancer therapies. Trends Cell Biol. 2018;28:723–37. 10.1016/j.tcb.2018.04.006 PMC6102047 29776716

[5] Cortesi M, Zanoni M, Pirini F, Tumedei MM, Ravaioli S, Rapposelli IG, et al. Pancreatic cancer and cellular senescence: tumor microenvironment under the spotlight. Int J Mol Sci. 2021;23:254. 10.3390/ijms23010254 PMC8745092 35008679

[6] Majewska J, Agrawal A, Mayo A, Roitman L, Chatterjee R, Sekeresova Kralova J, et al. p16-dependent increase of PD-L1 stability regulates immunosurveillance of senescent cells. Nat Cell Biol. 2024;26:1336–45. 10.1038/s41556-024-01465-0 PMC11321988 39103548

[7] Aasland D, Götzinger L, Hauck L, Berte N, Meyer J, Effenberger M, et al. Temozolomide induces senescence and repression of DNA repair pathways in glioblastoma cells via activation of ATR-CHK1, p21, and NF-κB. Cancer Res. 2019;79:99–113. 10.1158/0008-5472.CAN-18-1733 30361254

[8] Liu Y, Betori RC, Pagacz J, Frost GB, Efimova EV, Wu D, et al. Targeting telomerase reverse transcriptase with the covalent inhibitor NU-1 confers immunogenic radiation sensitization. Cell Chem Biol. 2022;29:1517–1531.e7. 10.1016/j.chembiol.2022.09.002 PMC9588800 36206753

[9] Passos JF, Nelson G, Wang C, Richter T, Simillion C, Proctor CJ, et al. Feedback between p21 and reactive oxygen production is necessary for cell senescence. Mol Syst Biol. 2010;6:347. 10.1038/msb.2010.5 PMC2835567 20160708

[10] Chan ASL, Zhu H, Narita M, Cassidy LD, Young ARJ, Bermejo-Rodriguez C, et al. Titration of RAS alters senescent state and influences tumour initiation. Nature. 2024;633:678–85. 10.1038/s41586-024-07797-z PMC11410659 39112713

[11] Serrano M, Lin AW, McCurrach ME, Beach D, Lowe SW. Oncogenic Ras provokes premature cell senescence associated with accumulation of p53 and p16INK4a. Cell. 1997;88:593–602. 10.1016/s0092-8674(00)81902-9 9054499

[12] Moiseeva O, Bourdeau V, Roux A, Deschênes-Simard X, Ferbeyre G. Mitochondrial dysfunction contributes to oncogene-induced senescence. Mol Cell Biol. 2009;29:4495–507. 10.1128/MCB.01868-08 PMC2725737 19528227

[13] Dörr JR, Yu Y, Milanovic M, Beuster G, Zasada C, Däbritz JH, et al. Synthetic lethal metabolic targeting of cellular senescence in cancer therapy. Nature. 2013;501:421–5. 10.1038/nature12437 23945590

[14] Herbig U, Jobling WA, Chen BP, Chen DJ, Sedivy JM. Telomere shortening triggers senescence of human cells through a pathway involving ATM, p53, and p21(CIP1), but not p16(INK4a). Mol Cell. 2004;14:501–13. 10.1016/s1097-2765(04)00256-4 15149599

[15] McConnell BB, Gregory FJ, Stott FJ, Hara E, Peters G. Induced expression of p16(INK4a) inhibits both CDK4- and CDK2-associated kinase activity by reassortment of cyclin-CDK-inhibitor complexes. Mol Cell Biol. 1999;19:1981–9. 10.1128/mcb.19.3.1981 PMC83991 10022885

[16] Sandhu C, Peehl DM, Slingerland J. p16INK4A mediates cyclin dependent kinase 4 and 6 inhibition in senescent prostatic epithelial cells. Cancer Res. 2000;60:2616–22. 10825132

[17] Sati S, Bonev B, Szabo Q, Jost D, Bensadoun P, Serra F, et al. 4D Genome rewiring during oncogene-induced and replicative senescence. Mol Cell. 2020;78:522–538.e9. 10.1016/j.molcel.2020.03.007 PMC7208559 32220303

[18] Correia-Melo C, Birch J, Fielder E, Rahmatika D, Taylor J, Chapman J, et al. Rapamycin improves healthspan but not inflammaging in nfκb1(-/-) mice. Aging Cell. 2019;18:e12882. 10.1111/acel.12882 PMC6351839 30468013

[19] James EL, Michalek RD, Pitiyage GN, de Castro AM, Vignola KS, Jones J, et al. Senescent human fibroblasts show increased glycolysis and redox homeostasis with extracellular metabolomes that overlap with those of irreparable DNA damage, aging, and disease. J Proteome Res. 2015;14:1854–71. 10.1021/pr501221g 25690941

[20] Braig M, Lee S, Loddenkemper C, Rudolph C, Peters AH, Schlegelberger B, et al. Oncogene-induced senescence as an initial barrier in lymphoma development. Nature. 2005;436:660–5. 10.1038/nature03841 16079837

[21] Chen Z, Trotman LC, Shaffer D, Lin HK, Dotan ZA, Niki M, et al. Crucial role of p53-dependent cellular senescence in suppression of Pten-deficient tumorigenesis. Nature. 2005;436:725–30. 10.1038/nature03918 PMC1939938 16079851

[22] Michaloglou C, Vredeveld LC, Soengas MS, Denoyelle C, Kuilman T, van der Horst CM, et al. BRAFE600-associated senescence-like cell cycle arrest of human naevi. Nature. 2005;436:720–4. 10.1038/nature03890 16079850

[23] Haferkamp S, Tran SL, Becker TM, Scurr LL, Kefford RF, Rizos H. The relative contributions of the p53 and pRb pathways in oncogene-induced melanocyte senescence. Aging. 2009;1:542–56. 10.18632/aging.100051 PMC2806033 20157537

[24] Raabe EH, Lim KS, Kim JM, Meeker A, Mao XG, Nikkhah G, et al. BRAF activation induces transformation and then senescence in human neural stem cells: a pilocytic astrocytoma model. Clin Cancer Res. 2011;17:3590–9. 10.1158/1078-0432.CCR-10-3349 PMC4086658 21636552

[25] Ewald JA, Desotelle JA, Wilding G, Jarrard DF. Therapy-induced senescence in cancer. J Natl Cancer Inst. 2010;102:1536–46. 10.1093/jnci/djq364 PMC2957429 20858887

[26] Chang BD, Broude EV, Dokmanovic M, Zhu H, Ruth A, Xuan Y, et al. A senescence-like phenotype distinguishes tumor cells that undergo terminal proliferation arrest after exposure to anticancer agents. Cancer Res. 1999;59:3761–7. 10446993

[27] Gewirtz DA, Holt SE, Elmore LW. Accelerated senescence: an emerging role in tumor cell response to chemotherapy and radiation. Biochem Pharmacol. 2008;76:947–57. 10.1016/j.bcp.2008.06.024 18657518

[28] Jun JI, Lau LF. The matricellular protein CCN1 induces fibroblast senescence and restricts fibrosis in cutaneous wound healing. Nat Cell Biol. 2010;12:676–85. 10.1038/ncb2070 PMC2919364 20526329

[29] Rottenberg S, Disler C, Perego P. The rediscovery of platinum-based cancer therapy. Nat Rev Cancer. 2021;21:37–50. 10.1038/s41568-020-00308-y 33128031

[30] Dasari S, Tchounwou PB. Cisplatin in cancer therapy: molecular mechanisms of action. Eur J Pharmacol. 2014;740:364–78. 10.1016/j.ejphar.2014.07.025 PMC4146684 25058905

[31] Perez EA. Microtubule inhibitors: Differentiating tubulin-inhibiting agents based on mechanisms of action, clinical activity, and resistance. Mol Cancer Ther. 2009;8:2086–95. 10.1158/1535-7163.MCT-09-0366 19671735

[32] Klein LE, Freeze BS, Smith AB 3rd, Horwitz SB. The microtubule stabilizing agent discodermolide is a potent inducer of accelerated cell senescence. Cell Cycle. 2005;4:501–7. 10.4161/cc.4.3.1550 15711127

[33] Dabrowska M, Skoneczny M, Uram L, Rode W. Methotrexate-induced senescence of human colon cancer cells depends on p53 acetylation, but not genomic aberrations. Anticancer Drugs. 2019;30:374–82. 10.1097/CAD.0000000000000731 30531292

[34] Song Y, Baba T, Mukaida N. Gemcitabine induces cell senescence in human pancreatic cancer cell lines. Biochem Biophys Res Commun. 2016;477:515–9. 10.1016/j.bbrc.2016.06.063 27311854

[35] Saleh T, Tyutyunyk-Massey L, Murray GF, Alotaibi MR, Kawale AS, Elsayed Z, et al. Tumor cell escape from therapy-induced senescence. Biochem Pharmacol. 2019;162:202–12. 10.1016/j.bcp.2018.12.013 30576620

[36] Nardella C, Clohessy JG, Alimonti A, Pandolfi PP. Pro-senescence therapy for cancer treatment. Nat Rev Cancer. 2011;11:503–11. 10.1038/nrc3057 21701512

[37] Wyld L, Bellantuono I, Tchkonia T, Morgan J, Turner O, Foss F, et al. Senescence and cancer: a review of clinical implications of senescence and senotherapies. Cancers. 2020;12:2134. 10.3390/cancers12082134 PMC7464619 32752135

[38] Gleason CE, Dickson MA, Klein Dooley ME, Antonescu CR, Gularte-Mérida R, Benitez M, et al. Therapy-induced senescence contributes to the efficacy of abemaciclib in patients with dedifferentiated liposarcoma. Clin Cancer Res. 2024;30:703–18. 10.1158/1078-0432.CCR-23-2378 PMC10870201 37695642

[39] Riviere-Cazaux C, Carlstrom LP, Neth BJ, Olson IE, Rajani K, Rahman M, et al. An untapped window of opportunity for glioma: targeting therapy-induced senescence prior to recurrence. NPJ Precis Oncol. 2023;7:126. 10.1038/s41698-023-00476-8 PMC10687268 38030881

[40] Ji J, Ding K, Cheng B, Zhang X, Luo T, Huang B, et al. Radiotherapy-induced astrocyte senescence promotes an immunosuppressive microenvironment in glioblastoma to facilitate tumor regrowth. Adv Sci. 2024;11:e2304609. 10.1002/advs.202304609 PMC11022718 38342629

[41] Saleh T, Carpenter VJ, Tyutyunyk-Massey L, Murray G, Leverson JD, Souers AJ, et al. Clearance of therapy-induced senescent tumor cells by the senolytic ABT-263 via interference with BCL-X(L) -BAX interaction. Mol Oncol. 2020;14:2504–19. 10.1002/1878-0261.12761 PMC7530780 32652830

[42] Beauséjour CM, Krtolica A, Galimi F, Narita M, Lowe SW, Yaswen P, et al. Reversal of human cellular senescence: roles of the p53 and p16 pathways. EMBO J. 2003;22:4212–22. 10.1093/emboj/cdg417 PMC175806 12912919

[43] Dirac AM, Bernards R. Reversal of senescence in mouse fibroblasts through lentiviral suppression of p53. J Biol Chem. 2003;278:11731–4. 10.1074/jbc.C300023200 12551891

[44] Lee S, Schmitt CA. The dynamic nature of senescence in cancer. Nat Cell Biol. 2019;21:94–101. 10.1038/s41556-018-0249-2 30602768

[45] Patel PL, Suram A, Mirani N, Bischof O, Herbig U. Derepression of hTERT gene expression promotes escape from oncogene-induced cellular senescence. Proc Natl Acad Sci USA. 2016;113:E5024–33. 10.1073/pnas.1602379113 PMC5003242 27503890

[46] Karabicici M, Alptekin S, Fırtına Karagonlar Z, Erdal E. Doxorubicin-induced senescence promotes stemness and tumorigenicity in EpCAM-/CD133- nonstem cell population in hepatocellular carcinoma cell line, HuH-7. Mol Oncol. 2021;15:2185–202. 10.1002/1878-0261.12916 PMC8334288 33524223

[47] Kadota T, Yoshioka Y, Fujita Y, Araya J, Minagawa S, Hara H, et al. Extracellular vesicles from fibroblasts induce epithelial-cell senescence in pulmonary fibrosis. Am J Respir Cell Mol Biol. 2020;63:623–36. 10.1165/rcmb.2020-0002OC 32730709

[48] Takasugi M, Okada R, Takahashi A, Virya Chen D, Watanabe S, Hara E. Small extracellular vesicles secreted from senescent cells promote cancer cell proliferation through EphA2. Nat Commun. 2017;8:15729. 10.1038/ncomms15728 PMC5467215 28585531

[49] Schwarzenbach C, Rinke J, Vilar JB, Sallbach J, Tatsch L, Schmidt A, et al. Therapy-induced senescence of glioblastoma cells is determined by the p21(CIP1)-CDK1/2 axis and does not require activation of DREAM. Cell Death Dis. 2025;16:357. 10.1038/s41419-025-07651-8 PMC12049523 40319068

[50] Coppé JP, Patil CK, Rodier F, Sun Y, Muñoz DP, Goldstein J, et al. Senescence-associated secretory phenotypes reveal cell-nonautonomous functions of oncogenic RAS and the p53 tumor suppressor. PLoS Biol. 2008;6:2853–68. 10.1371/journal.pbio.0060301 PMC2592359 19053174

[51] Faget DV, Ren Q, Stewart SA. Unmasking senescence: context-dependent effects of SASP in cancer. Nat Rev Cancer. 2019;19:439–53. 10.1038/s41568-019-0156-2 31235879

[52] Yang H, Wang H, Ren J, Chen Q, Chen ZJ. cGAS is essential for cellular senescence. Proc Natl Acad Sci USA. 2017;114:E4612–e4620. 10.1073/pnas.1705499114 PMC5468617 28533362

[53] Kang C, Xu Q, Martin TD, Li MZ, Demaria M, Aron L, et al. The DNA damage response induces inflammation and senescence by inhibiting autophagy of GATA4. Science. 2015;349:aaa5612. 10.1126/science.aaa5612 PMC4942138 26404840

[54] Hoare M, Ito Y, Kang TW, Weekes MP, Matheson NJ, Patten DA, et al. NOTCH1 mediates a switch between two distinct secretomes during senescence. Nat Cell Biol. 2016;18:979–92. 10.1038/ncb3397 PMC5008465 27525720

[55] Hernandez-Segura A, de Jong TV, Melov S, Guryev V, Campisi J, Demaria M. Unmasking transcriptional heterogeneity in senescent cells. Curr Biol. 2017;27:2652–2660.e4. 10.1016/j.cub.2017.07.033 PMC5788810 28844647

[56] Ruhland MK, Loza AJ, Capietto AH, Luo X, Knolhoff BL, Flanagan KC, et al. Stromal senescence establishes an immunosuppressive microenvironment that drives tumorigenesis. Nat Commun. 2016;7:11762. 10.1038/ncomms11762 PMC4899869 27272654

[57] Wiley CD, Velarde MC, Lecot P, Liu S, Sarnoski EA, Freund A, et al. Mitochondrial dysfunction induces senescence with a distinct secretory phenotype. Cell Metab. 2016;23:303–14. 10.1016/j.cmet.2015.11.011 PMC4749409 26686024

[58] Wang Z, Zhang Y, Wu F, Qiu B, Yin Y, Wang X, et al. Therapy-induced senescent glioblastoma cells sustain a procancer immune microenvironment by activating DDX58-mediated STAT1 signaling. Neuro Oncol. 2025;27:2265–80. 10.1093/neuonc/noaf107 PMC12526049 40259469

[59] Saul D, Kosinsky RL, Atkinson EJ, Doolittle ML, Zhang X, LeBrasseur NK, et al. A new gene set identifies senescent cells and predicts senescence-associated pathways across tissues. Nat Commun. 2022;13:4827. 10.1038/s41467-022-32552-1 PMC9381717 35974106

[60] Basisty N, Kale A, Jeon OH, Kuehnemann C, Payne T, Rao C, et al. A proteomic atlas of senescence-associated secretomes for aging biomarker development. PLoS Biol. 2020;18:e3000599. 10.1371/journal.pbio.3000599 PMC6964821 31945054

[61] Orjalo AV, Bhaumik D, Gengler BK, Scott GK, Campisi J. Cell surface-bound IL-1alpha is an upstream regulator of the senescence-associated IL-6/IL-8 cytokine network. Proc Natl Acad Sci USA. 2009;106:17031–6. 10.1073/pnas.0905299106 PMC2761322 19805069

[62] Acosta JC, Banito A, Wuestefeld T, Georgilis A, Janich P, Morton JP, et al. A complex secretory program orchestrated by the inflammasome controls paracrine senescence. Nat Cell Biol. 2013;15:978–90. 10.1038/ncb2784 PMC3732483 23770676

[63] Kastenhuber ER, Lowe SW. Putting p53 in context. Cell. 2017;170:1062–78. 10.1016/j.cell.2017.08.028 PMC5743327 28886379

[64] Bussian TJ, Aziz A, Meyer CF, Swenson BL, van Deursen JM, Baker DJ. Clearance of senescent glial cells prevents tau-dependent pathology and cognitive decline. Nature. 2018;562:578–82. 10.1038/s41586-018-0543-y PMC6206507 30232451

[65] Carreno G, Guiho R, Martinez-Barbera JP. Cell senescence in neuropathology: a focus on neurodegeneration and tumours. Neuropathol Appl Neurobiol. 2021;47:359–78. 10.1111/nan.12689 PMC8603933 33378554

[66] Hwang HJ, Kang D, Shin J, Jung J, Ko S, Jung KH, et al. Therapy-induced senescent cancer cells contribute to cancer progression by promoting ribophorin 1-dependent PD-L1 upregulation. Nat Commun. 2025;16:353. 10.1038/s41467-024-54132-1 PMC11699195 39753537

[67] Lai P, Liu L, Bancaro N, Troiani M, Calì B, Li Y, et al. Mitochondrial DNA released by senescent tumor cells enhances PMN-MDSC-driven immunosuppression through the cGAS-STING pathway. Immunity. 2025;58:811–825.e7. 10.1016/j.immuni.2025.03.005 40203808

[68] Kudo R, Safonov A, Jones C, Moiso E, Dry JR, Shao H, et al. Long-term breast cancer response to CDK4/6 inhibition defined by TP53-mediated geroconversion. Cancer Cell. 2024;42:1983. 10.1016/j.ccell.2024.10.013 39532066

[69] Milanovic M, Fan DNY, Belenki D, Däbritz JHM, Zhao Z, Yu Y, et al. Senescence-associated reprogramming promotes cancer stemness. Nature. 2018;553:96–100. 10.1038/nature25167 29258294

[70] Dimri GP, Lee X, Basile G, Acosta M, Scott G, Roskelley C, et al. A biomarker that identifies senescent human cells in culture and in aging skin in vivo. Proc Natl Acad Sci USA. 1995;92:9363–7. 10.1073/pnas.92.20.9363 PMC40985 7568133

[71] Georgakopoulou EA, Tsimaratou K, Evangelou K, Fernandez Marcos PJ, Zoumpourlis V, Trougakos IP, et al. Specific lipofuscin staining as a novel biomarker to detect replicative and stress-induced senescence. A method applicable in cryo-preserved and archival tissues. Aging. 2013;5:37–50. 10.18632/aging.100527 PMC3616230 23449538

[72] Ogrodnik M, Carlos Acosta J, Adams PD, d’Adda di Fagagna F, Baker DJ, Bishop CL, et al. Guidelines for minimal information on cellular senescence experimentation in vivo. Cell. 2024;187:4150–75. 10.1016/j.cell.2024.05.059 PMC11790242 39121846

[73] Tao W, Yu Z, Han JJ. Single-cell senescence identification reveals senescence heterogeneity, trajectory, and modulators. Cell Metab. 2024;36:1126–1143.e5. 10.1016/j.cmet.2024.03.009 38604170

[74] Narita M, Nũnez S, Heard E, Narita M, Lin AW, Hearn SA, et al. Rb-mediated heterochromatin formation and silencing of E2F target genes during cellular senescence. Cell. 2003;113:703–16. 10.1016/s0092-8674(03)00401-x 12809602

[75] Gurkar AU, Gerencser AA, Mora AL, Nelson AC, Zhang AR, Lagnado AB, et al. Spatial mapping of cellular senescence: emerging challenges and opportunities. Nat Aging. 2023;3:776–90. 10.1038/s43587-023-00446-6 PMC10505496 37400722

[76] Elyada E, Bolisetty M, Laise P, Flynn WF, Courtois ET, Burkhart RA, et al. Cross-species single-cell analysis of pancreatic ductal adenocarcinoma reveals antigen-presenting cancer-associated fibroblasts. Cancer Discov. 2019;9:1102–23. 10.1158/2159-8290.CD-19-0094 PMC6727976 31197017

[77] Neftel C, Laffy J, Filbin MG, Hara T, Shore ME, Rahme GJ, et al. An integrative model of cellular states, plasticity, and genetics for glioblastoma. Cell. 2019;178:835–849 e21. 10.1016/j.cell.2019.06.024 PMC6703186 31327527

[78] Coppé JP, Desprez PY, Krtolica A, Campisi J. The senescence-associated secretory phenotype: the dark side of tumor suppression. Annu Rev Pathol. 2010;5:99–118. 10.1146/annurev-pathol-121808-102144 PMC4166495 20078217

[79] Rando TA, Wyss-Coray T. Asynchronous, contagious and digital aging. Nat Aging. 2021;1:29–35. 10.1038/s43587-020-00015-1 PMC8248898 34223194

[80] Ravi VM, Will P, Kueckelhaus J, Sun N, Joseph K, Salié H, et al. Spatially resolved multi-omics deciphers bidirectional tumor-host interdependence in glioblastoma. Cancer Cell. 2022;40:639–655.e13. 10.1016/j.ccell.2022.05.009 35700707

[81] Yan L, Liang H, Qi T, Deng D, Liu J, He Y, et al. Senescence-specific molecular subtypes stratify the hallmarks of the tumor microenvironment and guide precision medicine in bladder cancer. BMC Cancer. 2025;25:297. 10.1186/s12885-025-13698-9 PMC11837361 39972258

[82] Zhang X, Huang Y, Li Q, Zhong Y, Zhang Y, Hu J, et al. Senescence risk score: a multifaceted prognostic tool predicting outcomes, stemness, and immune responses in colorectal cancer. Front Immunol. 2023;14:1265911. 10.3389/fimmu.2023.1265911 PMC10566297 37828981

[83] Hanahan D. Hallmarks of cancer: new dimensions. Cancer Discov. 2022;12:31–46. 10.1158/2159-8290.CD-21-1059 35022204

[84] Marin I, Boix O, Garcia-Garijo A, Sirois I, Caballe A, Zarzuela E, et al. Cellular senescence is immunogenic and promotes antitumor immunity. Cancer Discov. 2023;13:410–31. 10.1158/2159-8290.CD-22-0523 PMC7614152 36302218

[85] McHugh D, Durán I, Gil J. Senescence as a therapeutic target in cancer and age-related diseases. Nat Rev Drug Discov. 2025;24:57–71. 10.1038/s41573-024-01074-4 39548312

[86] Hasegawa T, Oka T, Son HG, Oliver-García VS, Azin M, Eisenhaure TM, et al. Cytotoxic CD4(+) T cells eliminate senescent cells by targeting cytomegalovirus antigen. Cell. 2023;186:1417–1431.e20. 10.1016/j.cell.2023.02.033 37001502

[87] Chambers ES, Vukmanovic-Stejic M, Shih BB, Trahair H, Subramanian P, Devine OP, et al. Recruitment of inflammatory monocytes by senescent fibroblasts inhibits antigen-specific tissue immunity during human aging. Nat Aging. 2021;1:101–13. 10.1038/s43587-020-00010-6 37118005

[88] Sagiv A, Burton DG, Moshayev Z, Vadai E, Wensveen F, Ben-Dor S, et al. NKG2D ligands mediate immunosurveillance of senescent cells. Aging. 2016;8:328–44. 10.18632/aging.100897 PMC4789586 26878797

[89] Arora S, Thompson PJ, Wang Y, Bhattacharyya A, Apostolopoulou H, Hatano R, et al. Invariant natural killer T cells coordinate removal of senescent cells. Med. 2021;2:938–50. 10.1016/j.medj.2021.04.014 PMC8491998 34617070

[90] Iltis C, Moskalevska I, Debiesse A, Seguin L, Fissoun C, Cervera L, et al. A ganglioside-based immune checkpoint enables senescent cells to evade immunosurveillance during aging. Nat Aging. 2025;5:219–36. 10.1038/s43587-024-00776-z PMC11839482 39730825

[91] Getu AA, Tigabu A, Zhou M, Lu J, Fodstad Ø, Tan M. New frontiers in immune checkpoint B7-H3 (CD276) research and drug development. Mol Cancer. 2023;22:43. 10.1186/s12943-023-01751-9 PMC9979440 36859240

[92] Yuan L, Tatineni J, Mahoney KM, Freeman GJ. VISTA: a mediator of quiescence and a promising target in cancer immunotherapy. Trends Immunol. 2021;42:209–27. 10.1016/j.it.2020.12.008 PMC8088836 33495077

[93] Rousseau A, Parisi C, Barlesi F. Anti-TIGIT therapies for solid tumors: a systematic review. ESMO Open. 2023;8:101184. 10.1016/j.esmoop.2023.101184 PMC10030909 36933320

[94] Ye J, Huang X, Hsueh EC, Zhang Q, Ma C, Zhang Y, et al. Human regulatory T cells induce T-lymphocyte senescence. Blood. 2012;120:2021–31. 10.1182/blood-2012-03-416040 PMC3437594 22723548

[95] Liu X, Mo W, Ye J, Li L, Zhang Y, Hsueh EC, et al. Regulatory T cells trigger effector T cell DNA damage and senescence caused by metabolic competition. Nat Commun. 2018;9:249. 10.1038/s41467-017-02689-5 PMC5770447 29339767

[96] Qiu Z, Li Z, Zhang C, Zhao Q, Liu Z, Cheng Q, et al. NK cell senescence in cancer: from molecular mechanisms to therapeutic opportunities. Aging Dis. 2025;17:1002–1033. 10.14336/AD.2025.0053 PMC12834417 40249925

[97] Colucci M, Sarill M, Maddalena M, Valdata A, Troiani M, Massarotti M, et al. Senescence in cancer. Cancer Cell. 2025;43:1204–1226. 10.1016/j.ccell.2025.05.015 40513577

[98] Braumüller H, Wieder T, Brenner E, Aßmann S, Hahn M, Alkhaled M, et al. T-helper-1-cell cytokines drive cancer into senescence. Nature. 2013;494:361–5. 10.1038/nature11824 23376950

[99] Dong Z, Luo Y, Yuan Z, Tian Y, Jin T, Xu F. Cellular senescence and SASP in tumor progression and therapeutic opportunities. Mol Cancer. 2024;23:181. 10.1186/s12943-024-02096-7 PMC11365203 39217404

[100] Huang L, Zhang C, Jiang A, Lin A, Zhu L, Mou W, et al. T-cell senescence in the tumor microenvironment. Cancer Immunol Res. 2025;13:618–32. 10.1158/2326-6066.CIR-24-0894 40232041

[101] Wang Z, Chen Y, Fang H, Xiao K, Wu Z, Xie X, et al. Reprogramming cellular senescence in the tumor microenvironment augments cancer immunotherapy through multifunctional nanocrystals. Sci Adv. 2024;10:eadp7022. 10.1126/sciadv.adp7022 PMC11529718 39485841

[102] Favaretto G, Rossi MN, Cuollo L, Laffranchi M, Cervelli M, Soriani A, et al. Neutrophil-activating secretome characterizes palbociclib-induced senescence of breast cancer cells. Cancer Immunol Immunother. 2024;73:113. 10.1007/s00262-024-03695-5 PMC11063017 38693312

[103] Sahin I, Zhang S, Navaraj A, Zhou L, Dizon D, Safran H, et al. AMG-232 sensitizes high MDM2-expressing tumor cells to T-cell-mediated killing. Cell Death Discov. 2020;6:57. 10.1038/s41420-020-0292-1 PMC7338458 32655895

[104] Amor C, Feucht J, Leibold J, Ho YJ, Zhu C, Alonso-Curbelo D, et al. Senolytic CAR T cells reverse senescence-associated pathologies. Nature. 2020;583:127–32. 10.1038/s41586-020-2403-9 PMC7583560 32555459

[105] Zhao W, Zhang Z, Xie M, Ding F, Zheng X, Sun S, et al. Exploring tumor-associated macrophages in glioblastoma: from diversity to therapy. NPJ Precis Oncol. 2025;9:126. 10.1038/s41698-025-00920-x PMC12048723 40316746

[106] Chen Z, Hambardzumyan D. Immune microenvironment in glioblastoma subtypes. Front Immunol. 2018;9:1004. 10.3389/fimmu.2018.01004 PMC5951930 29867979

[107] Gavish A, Tyler M, Greenwald AC, Hoefflin R, Simkin D, Tschernichovsky R, et al. Hallmarks of transcriptional intratumour heterogeneity across a thousand tumours. Nature. 2023;618:598–606. 10.1038/s41586-023-06130-4 37258682

[108] Wu J, Wang N. Current progress of anti‑PD‑1/PDL1 immunotherapy for glioblastoma (Review). Mol Med Rep. 2024;30:221. 10.3892/mmr.2024.13344 PMC11462401 39364736

[109] Liu M, Ji Z, Jain V, Smith VL, Hocke E, Patel AP, et al. Spatial transcriptomics reveals segregation of tumor cell states in glioblastoma and marked immunosuppression within the perinecrotic niche. Acta Neuropathol Commun. 2024;12:64. 10.1186/s40478-024-01769-0 PMC11036705 38650010

[110] Pudełek M, Ryszawy D, Piwowarczyk K, Lasota S, Madeja Z, Kędracka-Krok S, et al. Metabolic reprogramming of poly(morpho)nuclear giant cells determines glioblastoma recovery from doxorubicin-induced stress. J Transl Med. 2024;22:757. 10.1186/s12967-024-05541-9 PMC11318163 39135106

[111] Zanoni M, Sarti AC, Zamagni A, Cortesi M, Pignatta S, Arienti C, et al. Irradiation causes senescence, ATP release, and P2X7 receptor isoform switch in glioblastoma. Cell Death Dis. 2022;13:80. 10.1038/s41419-022-04526-0 PMC8786947 35075119

[112] Zhang C, Neha J, Zhang P, Dhaha X, Li SK, Mishra, et al. Aging and senescence: key players in brain tumor progression and drug resistance. Drug Resist Updat. 2025;81:101228. 10.1016/j.drup.2025.101228 40068246

[113] Venkataramani V, Tanev DI, Strahle C, Studier-Fischer A, Fankhauser L, Kessler T, et al. Glutamatergic synaptic input to glioma cells drives brain tumour progression. Nature. 2019;573:532–8. 10.1038/s41586-019-1564-x 31534219

[114] Woroniecka KI, Rhodin KE, Chongsathidkiet P, Keith KA, Fecci PE. T-cell dysfunction in glioblastoma: applying a new framework. Clin Cancer Res. 2018;24:3792–802. 10.1158/1078-0432.CCR-18-0047 PMC6095741 29593027

[115] Tomimatsu N, Di Cristofaro LFM, Kanji S, Samentar L, Jordan BR, Kittler R, et al. Targeting cIAP2 in a novel senolytic strategy prevents glioblastoma recurrence after radiotherapy. EMBO Mol Med. 2025;17:645–78. 10.1038/s44321-025-00201-x PMC11982261 39972068

[116] Liu B, Peng Z, Zhang H, Zhang N, Liu Z, Xia Z, et al. Regulation of cellular senescence in tumor progression and therapeutic targeting: mechanisms and pathways. Mol Cancer. 2025;24:106. 10.1186/s12943-025-02284-z PMC11963325 40170077

[117] Schmitt CA, Wang B, Demaria M. Senescence and cancer - role and therapeutic opportunities. Nat Rev Clin Oncol. 2022;19:619–36. 10.1038/s41571-022-00668-4 PMC9428886 36045302

[118] Peng N, Kang HH, Feng Y, Minikes AM, Jiang X. Autophagy inhibition signals through senescence to promote tumor suppression. Autophagy. 2023;19:1764–80. 10.1080/15548627.2022.2155794 PMC10262760 36472478

[119] Lin H, Liu C, Hu A, Zhang D, Yang H, Mao Y. Understanding the immunosuppressive microenvironment of glioma: mechanistic insights and clinical perspectives. J Hematol Oncol. 2024;17:31. 10.1186/s13045-024-01544-7 PMC11077829 38720342

[120] Gao Z, Jiang A, Li Z, Zhu L, Mou W, Shen W, et al. Heterogeneity of intratumoral microbiota within the tumor microenvironment and relationship to tumor development. Med Res. 2025;1:32–61.

[121] Troiani M, Colucci M, D’Ambrosio M, Guccini I, Pasquini E, Varesi A, et al. Single-cell transcriptomics identifies Mcl-1 as a target for senolytic therapy in cancer. Nat Commun. 2022;13:2177. 10.1038/s41467-022-29824-1 PMC9023465 35449130

[122] Tong J, He M. Identifying senescent cells as prognostic biomarkers and therapeutic targets of nasopharyngeal carcinoma by integrated analysis of single-cell and bulk-RNA sequencing data. Transl Cancer Res. 2025;14:5792–811. 10.21037/tcr-2025-659 PMC12554508 41158214

[123] Jing Z, Qiu B, Ai C, Wang C, Wang X, Li B, et al. Integrative analysis of single-cell and bulk transcriptome data reveals age-related immune cell alterations in primary glioblastoma associated with prognosis. Cancer Immunol Immunother. 2025;74:368. 10.1007/s00262-025-04206-w PMC12612434 41222641

